# Finding associations in a heterogeneous setting: statistical test for aberration enrichment

**DOI:** 10.1186/s13073-021-00864-4

**Published:** 2021-04-23

**Authors:** Aziz M. Mezlini, Sudeshna Das, Anna Goldenberg

**Affiliations:** 1 Harvard Medical School, Boston, USA; 2 Department of Neurology, Massachusetts General Hospital, Boston, USA; 3 Department of Computer Science, University of Toronto, Toronto, Canada; 4 Genetics and genome biology, Hospital for sick children, Toronto, Canada; 5 The Vector Institute, Toronto, Canada; 6 Evidation Health, Inc., San Mateo, CA USA; 7 CIFAR, Toronto, Canada

## Abstract

Most two-group statistical tests find broad patterns such as overall shifts in mean, median, or variance. These tests may not have enough power to detect effects in a small subset of samples, e.g., a drug that works well only on a few patients. We developed a novel statistical test targeting such effects relevant for clinical trials, biomarker discovery, feature selection, etc. We focused on finding meaningful associations in complex genetic diseases in gene expression, miRNA expression, and DNA methylation. Our test outperforms traditional statistical tests in simulated and experimental data and detects potentially disease-relevant genes with heterogeneous effects.

**Supplementary Information:**

The online version contains supplementary material available at (10.1186/s13073-021-00864-4).

## Background

Two-group statistical tests are widely used to characterize significant differences associated with an intervention or a condition. In a case/control setting, these tests can pinpoint variables of interest in the dataset analyzed. For example, gene expression data has been extensively used to characterize genes and pathways relevant to genetic diseases. If a gene is found to be differentially expressed (over-expressed or under-expressed) in the disease cases when compared to healthy controls, then it can potentially be associated with the disease. The differentially expressed gene can be causal for the disease, in which case it can become a candidate for therapeutic intervention, or the association it can be non-causal: for example a compensatory or a downstream consequence of the disease state itself (immune reaction, treatment effect, etc.). Nevertheless, finding the differentially expressed genes often generates candidates that are further tested for their mechanistic involvement in the disease [1–3]. The typical approach for finding differentially expressed genes relies on statistical tests (e.g., Limma [4]) that look for a broad pattern such as a global shift in mean expression between a target group (the cases) and a control group.

In this paper, we look for another mode of association that does not present as the typical broad pattern of mean difference typically targeted by the widely used statistical tests. In this mode, the considered variable will contain a significant number of outliers in cases (compared to controls), while the remaining majority of the cases will not be distinguishable from controls. To use the gene expression example again, consider the scenario where 10% of disease cases have an extremely low level of expression for a gene of interest but only 1% of the controls do. In the rest of the paper, we will call this hypothesized pattern of association “aberration enrichment” to distinguish it from the broad pattern of a mean/median/variance difference between two groups targeted by the currently used approaches. We will also describe the features/genes exhibiting this aberration enrichment pattern as “aberration enrichment features” or “features with heterogeneous effects”.

There are many reasons to believe this mode of aberration enrichment exists and is particularly relevant for the characterization of complex diseases. *First*, in complex diseases, it is expected that the disease causes would be spread across multiple genes, such that any particular gene would only be causal in a small proportion of the patients. This remains likely even when multiple different causal genes need to be hit to reach the disease state (as it is known to be the case in cancer [5]). It is unlikely in a complex disease to observe a single *causal* gene or factor that can broadly separate cases and controls. If we observe a single factor where the value for most patients differs from the typical value in healthy controls, then that factor is more likely to be a downstream consequence of the disease than to be causal. Otherwise, the disease would be mostly explained/caused by that one factor/gene contradicting the definition of a complex disease.

*Second*, work by major consortia have recently highlighted the importance of looking at rare events and outliers, rather than broad differences, to characterize disease biology. For example, work in the GTEX consortium [6] established links between being an outlier for a gene’s expression and having large impact rare cis-regulatory variants nearby. They also further linked the expression aberrations with diseases by selecting disease-associated variants and showing that they were highly enriched in variants predicted to generate expression outliers [6]. The pattern of aberration enrichment we are targeting in this paper corresponds to the expression outliers and could result from rare regulatory events involving SNVs, indels, and structural and epigenetic variants such as the ones investigated in the GTEX paper [6].

*Third*, there are many known genetic diseases where a portion of patients is explained by aberrant or outlier levels of a variable of interest such as a gene’s expression [7–10]. In the examples cited, a gene is associated with the disease through the presence of harmful coding variants in a proportion of patients. The authors observed that there were more patients without coding variants but whose expression levels for that gene are abnormally low. These expression aberrations were observed by manually counting the number of individuals with extremely low expression in a suspected causal gene. Providing a statistical test for automatically detecting aberration enrichment can further empower and formalize such analyses. Although these observations were mainly done in rare or Mendelian diseases where the causal gene is known through proven causal coding variation, the same mode of action (presence of expression aberrations/outliers) could also be relevant in more complex diseases.

The reasoning we made here for gene expression data also holds for other types of quantitative omics data where an enrichment in aberrations is a possible relevant pattern for disease association. This includes miRNA and noncoding RNA expression, protein expression, and DNA methylation.

In this paper, we present a new statistical test that aims at detecting novel associations through aberration enrichment: The presence of outlier values in a small but significant proportion of the cases. Outliers may be present in the data for many reasons including natural biological variability and technical artifacts and are not necessarily associated with a phenotype of interest. The focus here is not on outlier detection per se (such as in [11, 12]) but on finding consistent aberrations (in the same direction) that are significantly enriched in a subset of cases when compared to controls. Genes/factors discovered through this pattern of aberration enrichment can shed light on novel mechanisms and disease subtypes [13] undetected by previous methods looking for broad signals.

This pattern of aberration enrichment is discussed in the literature under other names. For example, OSACC [14] aims to identify signals that are present in a subset of the cases. They look for the best subset of individuals that leads to a stronger SNP association compared to taking all cases and all controls. In their case, the subset selection is guided and defined by a continuous known covariate variable, such as age (the context is finding G*E associations). In clinical trials’ literature, the pattern is known as heterogeneous treatment effects [15] where a drug could be working well in a subset of patients but still fail to show efficacy when considering all participants because the statistical methods used are looking for a mean effect. This has previously been discussed as “the trouble with the averages” [16]. Other related methods developed in 2005–2007 such as COPA [17] and Outlier-sum [18] rely on specific definitions of outliers and then look for an enrichment of these.

Using simulations, we show that our test is well calibrated and more powerful in detecting the aberration enrichment pattern compared to 11 other methods including widely used statistical tests, such as t-test and Limma, Wilcoxon, Levene, and Kolmogorov-Smirnov tests. We then use our test to examine 12 real/experimental datasets from GEO [19] spanning various cancers, neurodegenerative and auto-immune conditions, and 3 different data types (gene expression, miRNA expression, and DNA methylation). We discover new meaningful disease associations that were not captured by the traditional approaches.

Our test is available as a R package (aziztest) with usage examples: https://cran.r-project.org/web/packages/aziztest/index.html.

It can be installed using: install.packages(“aziztest”)

The rest of the code used to generate our results on simulations and experimental data can be found here [20]: https://github.com/azizmezlini/Aberration_Enrichment_Code.

## Methods

### Overview of our statistical test

The test presented in this paper is motivated by GSEA (Gene Set Enrichment Analysis) [21, 22]. GSEA takes a ranked list of genes and an annotated gene set (for example a pathway) and tests if the set is enriched at the top or bottom of the ranked list. An enrichment score is iteratively computed while walking through the ranked list. The score is incremented every time a positive gene (from the set) is encountered and is decremented every time a negative gene is encountered. The maximum enrichment score is saved and its significance is assessed by a permutation test.

In our test, we compute a ranked list of samples (cases and controls) using the measurement of interest (such as their expression levels for the gene being tested). Then we walk through the ranked list of samples, incrementing the enrichment score every time a case is encountered and decreasing it for every control. The increments and decrements are weighted by a standardisation of the measurement of interest **w**(absolute values of the *Z*-scores truncated at 0.5 minimum), therefore giving more weight to aberrations of larger scale.

The enrichment score at the *k*^*th*^ position is:

$$S_{k} = \frac{1}{n_{1}} \sum\limits_{i=1}^{k}{w_{i} X_{i}} - \frac{1}{n_{0}} \sum\limits_{i=1}^{k}{w_{i} \left(1-X_{i}\right)}, $$

where *n*_1_ and *n*_0_ are the total numbers of cases and controls, **w** is the vector of weights, and *X* is the indicator of being a case versus a control. For more details, see Additional file 1: Supplementary methods, where we give the definitions and equations.

We are interested in the maximum cumulative enrichment score in this iterative process. A large positive enrichment score is indicative of an enrichment of cases versus controls among the top of ranked samples in the list.

**Fig. 1:**
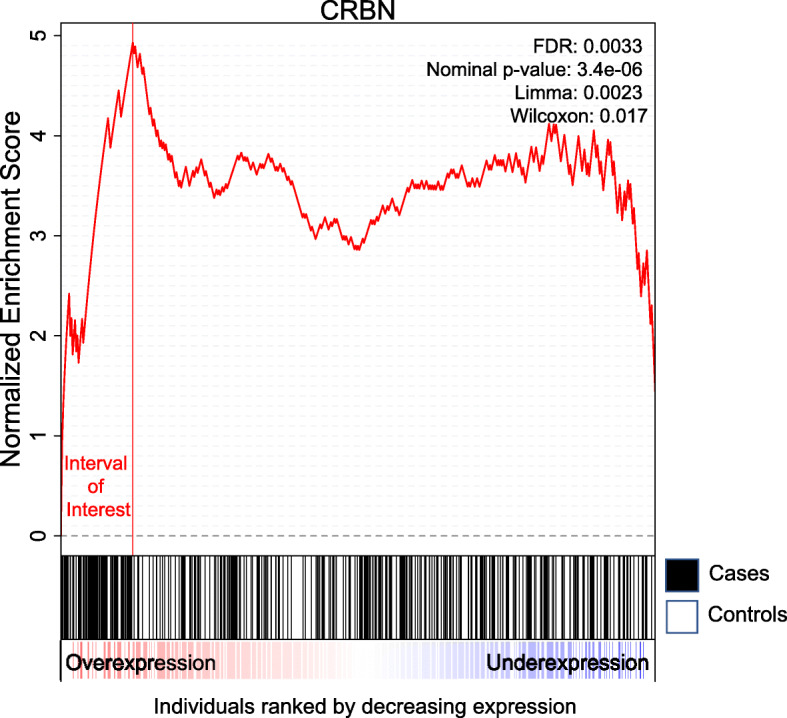
Example of a computed enrichment score. All individuals were ranked by decreasing expression of the *CRBN* gene. The *x* axis corresponds to the ranked individuals. In the second panel, cases correspond to black vertical bars and controls are white vertical bars. The enrichment score goes up whenever we encounter a case and goes down whenever we encounter a control. The maximum standardized score reached is 4.93 and corresponds to an uncorrected *p* value of 3.4E −06 and FDR of 0.003 for our test. There are 52 cases (19% of all cases) and 9 controls (4% of all controls) among the individuals to the left of the maximum. This was taken from the Alzheimer data in the “Alzheimer and Parkinson disease” section and it corresponds to the *CRBN* expression distribution. Note that Limma and Wilcoxon do not detect this gene as significant when simultaneously testing all 25,000 genes (uncorrected *p* values are respectively 0.002 and 0.01)

Additionally, under the null hypothesis with cases and controls uniformly ordered, the enrichment scores have a higher variance later down the walk (*S*_*k*_ can reach higher values by chance for higher *k*). This can introduce a positional bias and decrease the power of the test. We show that this variance can be analytically computed without approximations. Consequently, we can correct for the positional bias by adding a standardization step for the enrichment scores at every position (details and expanded equations in the Additional file 1: Supplementary methods). The maximum standardized enrichment score is taken and its significance is assessed with permutations. Figure 1 shows an example of a standardized enrichment score computed using CRBN gene expression levels on Alzheimer disease data (see the “Alzheimer and Parkinson disease” section).

Note that our test is looking for an enrichment of aberrations in a proportion of cases as compared to controls. It is therefore not symmetric in terms of case/control labels. Different associations might be detected if cases and controls are switched. This is not the problem in a setting where the focus is on identifying patterns associated with the cases. In a general two-group comparison, we can run our test in both directions.

### Experimental data analysis

We downloaded and analyzed several public datasets from GEO. This includes the following gene expression datasets: Alzheimer disease data GSE63063 [23], Parkinson disease data GSE99039 [24], inflammatory bowel disease data GSE73094 [25], heri. breast cancer data GSE47862 [26], and breast cancer metastasis data GSE48091 [27]. We also used the following miRNA expression datasets (breast cancer GSE73002 [28], ovarian cancer GSE106817 [29]) and DNA methylation datasets (Rheumatoid Arthritis GSE42861 [30], and schizophrenia datasets GSE74193 [31] and GSE80417 [32]).

After standardization, we preprocess each dataset by applying PEER [33] to remove known confounders (such as gender, age or batch if provided in the data) and 30 hidden factors (100 for the miRNA/methylation datasets which have more samples/features). Then, we test the residuals for differential expression analysis using our test and competing methods.

A full description of each dataset, including sample sizes, phenotype, tissue of origin, and covariates, is available in Additional file 1: Supplementary methods: Experimental data and preprocessing.

## Results

### Simulations

For our initial set of simulations, we start from Gaussian-simulated variables and then create an aberration enrichment pattern. We generate simulations by varying the following parameters:

- *n* = Sample size. Number of cases. We simulate *n* cases and *n* controls (here *n*=*n*_0_=*n*_1_. See Additional file 1: Figure S7 for the imbalanced setting).
- *r* = Proportion of the cases with an aberration in the considered gene.
- *m*,*s* = Mean and the standard deviation of the initial simulated Gaussian variable.
- *d* = Multiplier controlling the magnitude of the aberration. The proportion *r* of affected individuals will have their average expression shifted *d*×*s* away from the rest of the cases and controls.

**Fig. 2:**
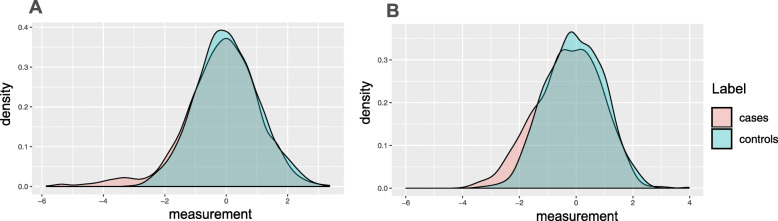
Examples of considered scenarios for aberration enrichment pattern. *r* controls the proportion of individuals affected among the cases and *d* is the magnitude of the effect within those individuals. **a** *r*=0.05, *d*=3, *n*=500**b** *r*=0.15, *d*=1.5, *n*=500. Note that the aberration enrichment pattern often appears as a heavier tail for cases rather than a secondary cluster of cases especially for lower values of *r*. Intuitively, *d* affects the location of the red area relative to the mean of the distribution while *r* more specifically affects the size of the red area

Simulations provide a controlled setting to assess the validity and power of our test in comparison to the widely used parametric and non-parametric approaches such as *t*-test and Wilcoxon, Levene, and Kolmogorov-Smirnov tests. The pattern of aberration enrichment simulated here could still be captured by traditional methods testing for a shift in mean or variance between cases and controls. We first simulate a variable with an aberration enrichment pattern of association by sampling the cases and controls from a Gaussian (our test makes no Gaussian assumptions. Similar results with other non-Gaussian distributions are included in the Additional file 1: Figure S8) and then perturbing a proportion *r* of the cases. The perturbation is a shift by *d* times the standard deviation in one selected direction (an increase or a decrease). Figure 2 is an example of what the simulated data looks like given different parameter choices.

We then tested the ability to detect these introduced aberration enrichment signals for our and the other parametric and non-parametric tests. Varying the sample sizes and the simulations parameters, we assess how often each method is able to detect the association given a nominal *p* value threshold of 0.05 or a more stringent Bonferroni threshold of 2×10^−6^ (typically used in gene expression analyses to correct for multiple hypothesis testing). In these simulations, we are generating one variable at a time and are assessing the power to detect that true association. In Additional file 1: Figures S6-S8, we verify that our test has no false positives in these experiments. Additionally, type-1-error of the different methods is discussed in the next section in a full realistic simulation setting with large numbers of associated and non-associated variables and again later on experimental data (for example in Fig. 7d). For every set of simulation parameters, we repeat the experiment 200 times. The signal is considered detected if the *p*-value is less than the chosen threshold in more than half of the repeats.

#### Power comparison

**Fig. 3:**
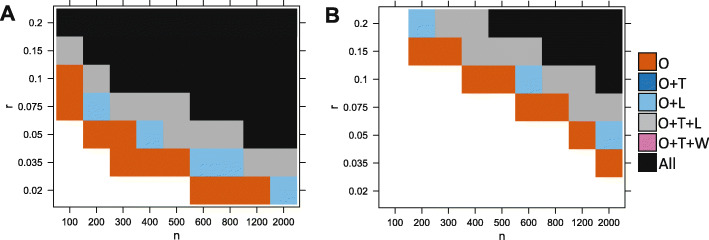
Ability of the different tests to detect the association, depending on simulations parameters *n* (sample size) and *r* (proportion of affected cases). In **a**, a nominal *p* value threshold of 0.05 is used. In **b**, a lower *p* value threshold of 2×10^−6^ was used to mimic a realistic data analysis scenario where correction for multiple tests is required. We compare our test (O), the Levene test (L), *t*-test (T), and Wilcoxon (W). A method is able to detect the signal if the *p* value is lower than the threshold in the majority of 200 reruns. Here we show the results for *d*=3. White is for the set of experiments where no method detected the signal, vermillion (red-orange) is when only our test detected the signal, light blue is when our test and the Levene test both detected it, gray is when our method, the *t*-test and the Levene test detected it and black is when all considered methods can detect the signal (including the Wilcoxon test)
- When the proportion *r* and the sample size *n* are large, the number of cases with aberrations is high enough to create a significance shift in the mean (or variance) of the distribution of the cases compared to the distribution of the controls. In this case, most methods are able to detect the differential expression (black/ gray/ blue).
- Between these two regions, there is a domain where only our test is powerful enough to detect the association due to aberration enrichment (vermillion-red region).

Figure 3 shows that variation in parameter space leads to three distinct outcomes.

- When either the proportion *r* or the sample size *n* is too small, no test can detect the association. The number of cases with an observable aberration is too low to generate enough statistical power (white region).

**Fig. 4:**
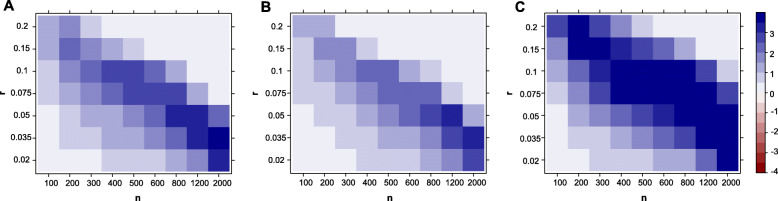
Comparison of the *p* value magnitude between our test and **a***t*-test test, **b** Levene test, **c** Wilcoxon test, depending on the simulation parameters *n* (sample size) and *r* (proportion of affected cases). Here we show the results for *d*=3. The colors indicate the difference in log10 between the *p* values returned by both method. For example **b** indicates that our test’s *p* value is two orders of magnitude smaller (×10^−2^) than that of the Levine test. We capped the maximal difference at 4 for visual clarity. We ran 10^8^ permutations to compute the *p* values for our test; therefore, we set the minimal *p* value to 10^−7^ for all methods in order to avoid artifacts of *p* values estimation accuracy

Overall, we found that our test performs best, followed by the Levene test and the *t*-test. Wilcoxon had a lower power than the*t*-test, and finally, the Kolmogorov-Smirnov test was the least powerful (Kolmogorov-Smirnov test results not shown here for clarity purposes. See Table 1). The Levene test performed slightly better on average than the *t*-test in this context (with *d*=3). Figure 4 further shows there is a large difference in power between our test and the other tests in terms of magnitude of *p* values, which was often several orders of magnitude lower for our test. This is especially true for the lower values of *r* (the signal is present in a smaller proportion of patients). For example, when *r*≤0.05 the *p* values returned by our test are often more than 4 orders of magnitude (10,000 times) smaller than those returned by any other methods.

**Table 1:** Comparison of the false-positive rate and the power of our test, and 11 other approaches and statistical tests. The average performance over 1000 simulations is shown here

	Power				False discovery rate			
Sample size *n*	200	300	400	600	200	300	400	600
Our test	**0.072**	**0.297**	**0.651**	**0.838**	0.095	0.092	0.084	0.087
COPA75	0.016	0.039	0.107	0.202	0.081	0.079	0.1	0.084
COPA9	0.033	0.109	0.312	0.511	0.064	0.071	0.076	0.09
COPA95	0	0.076	0.251	0.429	0	0.055	0.079	0.075
Outlier-sum	0.038	0.172	0.418	0.618	0.063	0.083	0.093	0.099
Wilcoxon	0.006	0.016	0.053	0.111	0.104	0.087	0.089	0.099
Kolmogorov-Smirnov	0.002	0.007	0.024	0.052	0.078	0.086	0.091	0.077
Logistic regression	0.01	0.036	0.156	0.34	0.062	0.074	0.065	0.092
ANOVA	0.01	0.036	0.156	0.34	0.062	0.074	0.065	0.092
Limma	0.016	0.046	0.172	0.358	0.09	0.091	0.079	0.103
*t*-test	0.016	0.045	0.172	0.357	0.091	0.09	0.079	0.102
Levene	0.007	0.03	0.118	0.256	0.086	0.086	0.091	0.11
Fisher Combination t+L	0.053	0.189	0.548	0.792	0.228	0.205	0.184	0.176
*K*-Means+ chi-squared	0.003	0.006	0.021	0.04	0.078	0.08	0.065	0.079

**Fig. 5:**
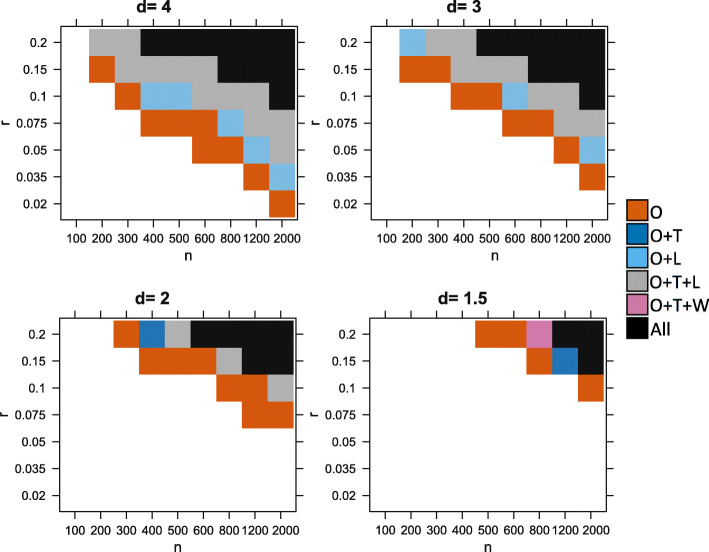
Ability of different tests to detect the simulated association, depending on simulations parameters *n* (sample size), *r* (proportion of affected cases), and *d*. *p* value threshold of 2×10^−6^. A method is able to detect the signal if the *p* value is lower than the threshold in the majority of 200 reruns. We compare our test (O), the Levene test (L), *t*-test (T), and Wilcoxon (W) test

We also explored different scenarios by varying the other simulation parameters. We found that changing the mean *m* or variance *s* of the Gaussian had no effect on performance. In contrast, changing the value of the *d* parameter (perturbation magnitude multiplier) had a clear effect on performance in Fig. 5. Higher values of *d* meant more cases became clear outliers for the expression of the considered gene, which makes the aberration enrichment signal easier to detect for our test. Higher *d* also means a higher effect on the overall mean/variance of the cases; therefore, the power increases for all methods. Inversely, lower values of *d* negatively affect the performance of all methods. The ordering of the methods is overall maintained across the values of *d* with our test having the best performance in all scenarios followed by Levene/*T*-test and then Wilcoxon. However, we observe that the lower values of *d* are more severely affecting the Levene test compared to other methods. For *d*≤2, the *t*-test start outperforming the Levene test (dark blue instead of light blue). This is expected because higher magnitude of perturbations have a larger effect on the variance.

**Fig. 6:**
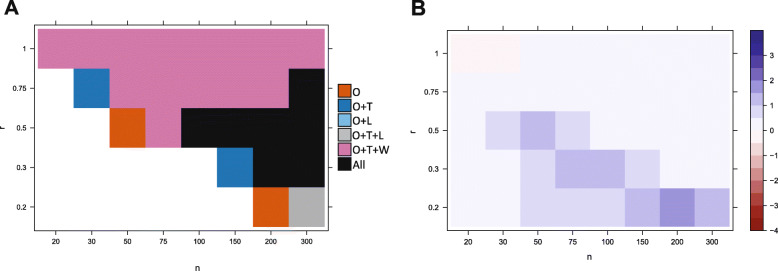
Results under less heterogeneity, i.e. higher values of *r.***a** Ability of the different tests to detect the association for higher values of *r*. A method is able to detect the signal if the *p* value is lower than the threshold in the majority of 200 reruns. Here we show the results for *d*=3. We compare our test (O), the Levene test (L), *t*-test (T), and Wilcoxon (W) test. **b** Comparison of the *p* value magnitude between our test and the best out of*t*-test, Levene test, and Wilcoxon test, depending on simulations parameters *n* (sample size) and *r* (proportion of affected cases). Here we show the results for *d*=3. The colors indicate the difference in log10 between the *p* values returned by both method. For example **b** indicates that our test’s *p* value is two orders of magnitude (100 times) smaller than that of the Levine test. We capped the maximal difference at 4 for visual clarity

Next, we studied the limitations of our test by increasing the values of *r*. For *r*=1, there is no heterogeneity, i.e., all cases are affected the same way, while lower values of *r* correspond to the aberration enrichment (or heterogeneous response) scenario targeted by our test. Figure 6 shows that our test continues to be more powerful up to *r*≤0.5 with a large difference in *p* value magnitude for *r*≤0.3. For larger values of *r*≥0.7, there is no longer an advantage over using a *t* test. We observed that the Levene test performance drops dramatically for the higher values of *r* and can no longer find the signal that is detectable by all other methods (pink region). For *r*=1, we are essentially simulating cases that are mean shifted from the controls with no effect on the variance.

Other scenarios, such as very low values of *r* (Additional file 1: Figure S1) and lower values of *d*≤1 (Additional file 1: Figure S2 and S3), settings with imbalanced number of cases and controls (Additional file 1: Figure S7), and other non-Gaussian distributions (Additional file 1: Figure S8), can be found in the supplementary materials. Overall, we conclude that our test can be a powerful alternative to currently used methods for scenarios with *d*≥0.7 and *r*≤0.5. Large gain in statistical power are obtained especially in settings with *d*≥1.5 and *r*≤0.3, meaning that less than 30% of the case group are different from the controls. We recommend using our test when heterogeneity is suspected.

#### Simulating gene expression data

In the previous section, we showed that our test was more powerful than a *t*-test, Wilcoxon test, and Levene test to detect the aberration enrichment pattern under the simplistic assumption of perturbed Gaussian when simulating a single variable at a time. In the differential expression literature, there are approaches that work on genome-wide expression datasets (instead of a single gene) and that are more powerful than a *t*-test [34] for finding differentially expressed genes. Here we generated simulations based on real/experimental microarray expression data. The goal is to assess the performance of our test (power and type-1-error control) in comparison to other methods in the realistic setting typically considered when analyzing differential expression. By perturbing only a subset of the genes (ground truth positives), we can also assess whether the different methods are well calibrated by analyzing the rate of false positives.

This setting also allows us to compare to the widely used method Limma [4] that is applicable to genome wide expression datasets. Limma uses empirical Bayes to borrow information across genes in order to empower the detection of differential expression, especially for lower sample sizes. Its efficiency has been proven in methods reviews publications where it always showed better or on par power and false-positive control compared to all state-of-the-art methods, for both microarray [34, 35] and RNA-Seq experiments [36, 37]. We also compare to several widely used approaches and statistical tests including *t*-test, Wilcoxon test, Levene test, ANOVA, logistic regression, and the Kolmogorov-Smirnov test.

Moreover, two less widely used approaches were developed in 2005–2007 with the exact same aim as our test: detecting signal that is present in only some of the cases. Both tests rely on predefined definitions of outliers and look for an unusual enrichment of outliers in the cases. COPA [17] uses specific quantiles in the cases as the test statistic (0.75, 0.9, 0.95), while Outlier-sum [18] defines outliers using the interquartile range and then takes the sum of the outliers as the test statistic. In both methods, case/control label permutations are used to assess significance.

We use the 238 healthy controls from GSE63063 [23] from the Gene Expression Omnibus (GEO). The dataset contains 25,549 features/genes. We created our dataset by sampling gene expression from the gene expression data and adding Gaussian noise *ε*∼*N*(0,*δ*^2^) for a simulated *n* cases and *n* controls. We repeat the simulation 1000 times while downsizing the data to 1000 random genes in every simulation for efficiency purposes. We randomly selected a set of *g* genes as disease-associated among the 1000 genes in the data. The controls were left untouched but a proportion *r* of the cases were perturbed in every disease-associated gene, similarly to what we did in the “Power comparison” section (the expression values for the considered gene and the selected cases, were shifted in one direction by a factor of *d* times the standard deviation of the gene). We fixed the simulation parameters to *r*=0.1, *d*=2, *δ*=0.01, *g*=10 and varied the sample size *n*. We ran our method and 11 other tests on each simulated dataset and for every choice of sample size, then we assessed the type 1 error and the power of each method. More specifically, we measured the false-positive rate using an FDR threshold of 0.1 and the true-positive rate (proportion of the true genes that were detected).

Table 1 shows that our test outperforms all other methods with a large margin similarly to what we observed in the previous section. Across the 1000 simulation reruns, the power of our test is always significantly superior: *p* values ≤2.7×10^−6^ in a pairwise comparison with every other method (*t*-test). Almost all approaches are well calibrated and do not show inflation for type-1 errors. Given that we used *FDR*≤0.1 as the criterion for determining positive calls in every simulation, we expect the false discovery rate to be very close or lower than 0.1 for all methods.

In Table 1, applying Limma or *t*-test results in very similar *p*-values and an equivalent performance between these two methods. It seems that borrowing information across genes might not be helping Limma to noticeably improve performance over the *t*-test, for the sample sizes considered *n*≥200 and in the setting of an aberration enrichment pattern. Additionally, we verified that using a logistic regression or ANOVA results in *p* values that are equivalent to the *t*-test *p* values in this setting.

Our test is more adapted to the detection of aberration enrichment pattern than Limma, *t*-test or the Levene test, confirming our previous conclusion that when the signal is detectable, there is a considerable difference in power between our test and existing statistical tests and differential expression methods. This performance gap becomes even wider for smaller values of *r* (proportion of affected cases) as shown in Additional file 1: Figure S4, where we repeated the same experiment with *r*=0.05. This is expected given that there will be less of an impact on the mean or variance of the cases’ distribution when fewer individuals show aberrant levels for the gene of interest, therefore giving a larger advantage to our test since it does not rely on detectable broad differences between all cases and controls.

Comparing our test to the other methods that were also designed to detect the aberration enrichment pattern (COPA[17] and outlier-sum [18]), we observe a better performance for our test. Unlike the other methods, we do not rely on the choice of thresholds or on strict definition of outliers. Individuals can still contribute to the enrichment even when they are not clear outliers on their own. This distinction results in an even larger gap in performance between our method and these outlier-based methods when the effect sizes are smaller (*d*≤1.5) and less affected cases present as clear outliers. In Additional file 1: Table S7, we run the same experiment with a slightly lower *d*=1.5. Our test still performed best compared to all methods, while the performance of Outlier-sum and COPA deteriorated to below the performance of a *t*-test. Furthermore, the test statistics used in COPA and Outlier-Sum makes little use of controls and are affected by how heavy-tailed the data distribution is (see Additional file 1: Figure S8). Therefore, we observed that comparing or ranking test statistics (as proposed in [17]) is actually not informative of which variables are going to be most significant. Computationally expensive case-control label permutations on every tested variable are the only way to assess whether a test statistic correspond to a possible association.

We also compared to the baseline of using a clustering algorithm on the data (here *K*-Means with *K*=2), followed by a chi-squared test. Clustering is an unsupervised approach for describing the heterogeneity in the data and can be an alternative approach to identify a subgroup of cases that are affected corresponding to the aberration enrichment pattern. Our simulated data corresponds in fact to two distributions: (1) the controls and unaffected cases that are sampled with noise from the original gene expression distribution and (2) affected cases that were perturbed afterward by a shift of *d*∗*s*. Nevertheless, this clustering baseline did not perform well in this scenario. This is expected as in our simulations the affected cases do not necessarily form a visually separable cluster on their own. As illustrated in the example of Fig. 2, the affected cases often correspond to a heavier tail rather than a separate mode in the distribution.

Finally, we used a joint test of scale and location by combining both *t*-test and Levene test with the Fisher’s method as proposed in [38] (Fisher Combination). Combining *t*-test (or Limma) and the Levene test in one joint scale-location test gives higher power than either test used separately as shown in Table 1. However, the joint test is not well calibrated in this setting as illustrated by the high false-positive rates limiting its applicability in an experimental data setting. Moreover, our test is still more powerful than the combined test.

We conclude that our test is indeed well calibrated and that it is significantly more powerful than current tests to detect true associations when the signal takes the form of an aberration enrichment rather than a global shift in mean. Among the well-calibrated approaches, Outlier-Sum and COPA came second and third in performance, beating the methods that are looking for a mean difference (*t*-test, Limma, ANOVA, Logistic regression), a variance difference (Levene), or a median difference (non-parametric Wilcoxon).

### Results on experimental data across diseases

We downloaded, preprocessed and analyzed case-control gene expression datasets from Gene Expression Omnibus (GEO). The sample sizes for each dataset are summarized in Additional file 1: Table S1. The preprocessing involved removing 30 hidden (latent) factors with PEER [33] as was done in GTEX study on rare expression aberrations [39]. Since the simulation showed that Limma was as good or better than *t*-test, we ran Limma and our statistical test for each of our experimental data studies and analyzed the genes detected by each method. Our results on Wilcoxon test can be found in the Additional file 1: Table S6. As we can see in Table 2, there was a number of differentially expressed genes that were detected by both methods. In this analysis, we focus on the novel genes that were only found by our test. Additionally, whenever an association is found by our test, we can identify which subgroup of individuals and which interval of aberrant expression contributed to the test statistic and compute an estimated value of *r* (proportion of cases affected) for that gene (see Additional file 1: Supplementary methods).

**Table 2:** Experimental data findings: number of genes detected

Threshold	Bonferroni			FDR <0.1		
Method	Our test	Limma	∩	Our test	Limma	∩
Alzheimer vs ctr	23	25	19	69	100	50
Parkinson	0	0	0	0	0	0
CD	2	0	0	2	2	1
UC	1	0	0	1	2	0
IBD inflammation	8	0	0	49	0	0
Breast cancer	15	9	6	406	224	118
Breast metastasis	2	0	0	4	0	0

#### Alzheimer and Parkinson disease

**Fig. 7:**
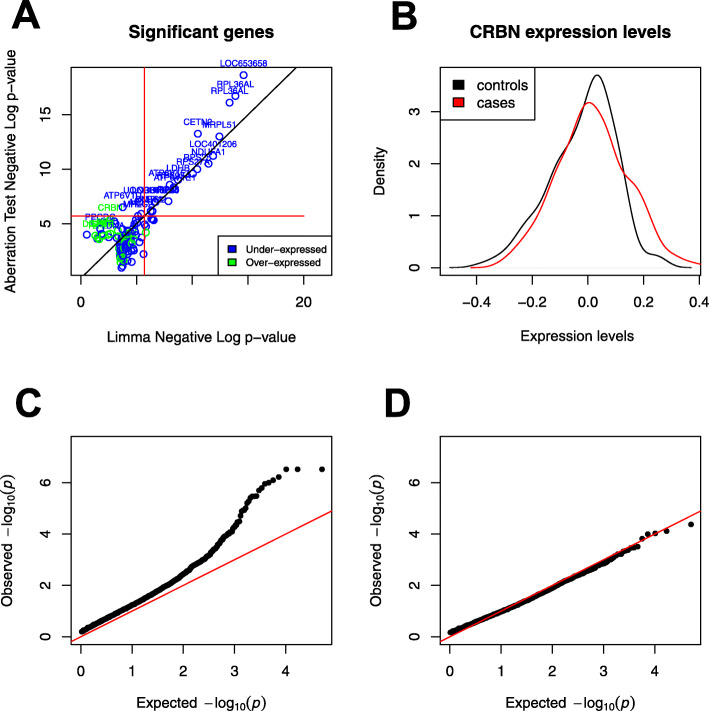
Results on Alzheimer’s disease. **a** Genes differentially expressed or aberration enriched in Alzheimer versus healthy controls, discovered using our test versus Limma. The genes that have *FDR*≤0.1 for either method are plotted. Gene names are added for all the significant genes by our method and the top 10 heterogeneous associations (*r*≤0.3). Red lines correspond to the Bonferroni significance threshold. **b** Distribution of expression levels in cases and controls for the gene *CRBN* discovered by our test. **c** QQ plot of the *p* values returned by our test on the Alzheimer data. **d** QQ plot of the *p* values returned by our test after randomly permuting the samples

The GEO dataset GSE63063 [23] contains gene expression of 284 Alzheimer disease patients (AD), 189 mild cognitive impairment patients (MCI), and 238 healthy matched controls measured in blood. We ran Limma and our test to find genes that are differentially expressed between Alzheimer patients and healthy individuals. In this analysis, we want to find novel genes that would not be picked up by Limma or genes that are much more significant by our test. Those genes would be above the diagonal in Fig. 7a. We observe that genes such as *UQCRH*, *ATP6V1D*, *CRBN*, *POMP*, and *EIF3E* fit this criterion with the first two below Bonferroni significance threshold and the last three with FDR <0.01. *UQCRH* is part of the KEGG pathway for Alzheimer disease, listed in both the organism specific and the conserved biosystems. *CRBN* or Cereblon (*FDR*=0.003) is known to play a role in memory and learning and it has been previously associated with mental retardation [40]. It is also used in ubiquitination/proteasomal degradation of Tau [41] which could be relevant for Alzheimer disease [42]. *MYL6* only reaches significance by using our test (Bonferroni-corrected) while being under the significance threshold using Limma.

The top 10 aberration enrichment genes (which exhibit a highly heterogeneous signal corresponding to an estimated *r*<0.3), with *FDR*<0.1 by our test, are *CRBN* (ILMN1668582), *PPCDC*, *FBP1*, *DDX17*, *SYT13*, *GPER*, *DISC1*, *LRP3*, *TLR2*, and *DNAJA1*. Even though the expression levels were measured in blood, at least 4 of the genes found are known for their functions in the brain (GPER, DISC1, TLR2, DNAJA1). Furthermore, at least 2 genes have been shown to be involved in Alzheimer disease in the literature. For example, *DNAJA1* mediates Tau clearance [43] and *TLR2* is a major receptor for Alzheimer’s A *β* with a proven role in activating neuroinflammation [44]. These novel associations would not be detectable by other methods, showing the importance of going beyond differential expression and looking for heterogeneous effects and the aberration enrichment pattern.

The majority of associations uncovered in Fig. 7a are in the form of an under-expression of the considered gene in Alzheimer patients. Exceptions to this are *CRBN*, *DDX17*, *GPER*, *DISC1*, *LRP3,* and *TLR2*. Note that *DISC1* and *TLR2* are on the DisGenet [45] Late onset Alzheimer disease gene set, but that we found no significant enrichment using DisGenet for any method (our test, Limma, Wilcoxon). This is unsurprising, given that the purpose of our method is to find novel heterogeneous associations that are hard to discover with previous approaches and that the curated datasets put more focus on associations made on Single Nucleotide Polymorphisms data rather than large-scale gene expression data we are using here.

In Fig. 7b, we plotted the distribution of *CRBN* expression levels in cases and controls to better show the nature of the association, where a subgroup of cases have aberrant overexpression of the gene. Figure 7c shows that there could be a large number of genes exhibiting some aberration enrichment signal in association with Alzheimer. Comparing the QQ plot in Fig. 7c to the one in Fig. 7d where we permuted the labels confirms that the associations discovered are not spurious hits due to a badly calibrated statistical test but signals that are truly associated with the case-control labels.

We performed a similar analysis of Parkinson disease (IPD) and found no associated genes using any of the considered methods. The dataset (GSE99039 [24]) consisted of whole blood gene expression data for 205 IPD cases and 233 controls.

#### Inflammatory bowel disease

The inflammatory bowel disease data (GSE73094 [25]) contains the gene expression of 712 pre-selected genes, including 440 genes in IBD GWAS risk loci and 15 housekeeping genes, in 608 samples from Crohn’s disease (CD) patients, 331 from Ulcerative Colitis (UC) patients, and 50 samples from non-IBD individuals. The samples were taken from the colon and terminal ileum. Overall, 374 of the samples were taken during inflammation and 609 taken from non-inflamed tissues (6 samples with missing inflammation status were removed).

We first looked for genes associated with CD versus UC and vice-versa by considering the not inflamed samples which were more numerous than the inflamed ones. This resulted in 181 UC and 314 CD non-inflamed samples. After preprocessing (see Additional file 1: Supplementary methods), we looked for genes associated with CD and genes associated with UC by comparing each group to the other.

Only one gene was significantly associated with UC. *C11orf9* was detected by our test. The gene is also called *MYRF* and was previously mentioned in the IBD literature as part of a co-expression cluster of upregulated genes [46] and the nearby SNP rs4246215 was previously associated with IBD in GWAS [47, 48].

Two genes were found to be significant for CD: *BTNL2* and *IRF4*. Both were detected as significant only with our test (Bonferroni and FDR). *IRF4* was discovered by Limma too with *FDR*=0.09 (*FDR*=6*E*−4 by our test). *BNTL2* was not a broad effect (*r*=0.15), and therefore, it was not detected by other methods.

We also looked for genes associated to inflammation status across all conditions by taking all samples from the original data (374 inflamed and 609 non inflamed) and correcting for disease type as a confounder.

**Fig. 8:**
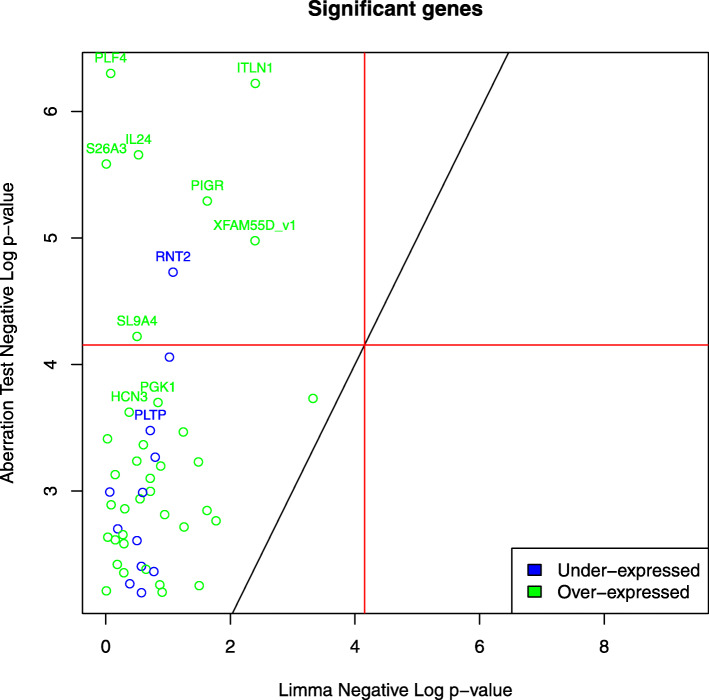
Gene expression associations in IBD, inflamed versus non-inflamed samples, discovered using our test versus Limma. The genes that have FDR ≤0.1 for either method are plotted. Gene names are added for all the significant genes by our method and the top 10 heterogeneous associations (*r*≤0.3). Red lines correspond to the Bonferroni significance threshold

In Fig. 8, 8 genes were found to be significantly associated with inflammation: *PLF4*, *ITLN1*, *IL24*, *S26A3*, *PIGR*, *RNT2*, *SL9A4,* and *FAM55D*. All of them were only detected with our test.

#### Breast cancer

The heritable breast cancer data (GSE47862 [26]) contains gene expression in peripheral blood in 158 women with heritable breast cancer and 163 healthy controls. The top 3 associated genes by our test were Entrez-id 100129342, *PIK3C2B,* and *CR2*. We identified a large number of associated genes with heterogeneous effects (estimated *r*<0.3). The top 10 genes of our gene list are *PSIP1*, *SLCO2B1*, *TLX3*, *CDKAL1*, *MCMDC2*, *GPAA1*, *B4GALT1*, *FUT4*, *PIGR*, and *CDCA7* (*FDR*<0.025). Most of these genes (9/10) have substantial evidence in the literature of their involvement in breast cancer. For example, it is known that silencing *CDCA7* in triple-negative breast cancers reduced tumorigenicity and invasion in [49], while the forced expression of *GPAA1* in [50] was shown to increase them. PSIP1 has also been shown to directly promote tumorigenicity in breast cancer [51]. The expression of *SLCO2B1* was shown to be significantly correlated with histological grade in ER+ breast cancer [52]. *TLX3* is also known as T Cell Leukemia Homeobox 3 and is a transcription factor oncogene. rs9368197 in an intron of *CDKAL1* is associated with breast cancer risk [53]. Knock-down of the *B4GALT1* gene and the inhibition of its function has been shown to inhibit the estrogen-induced proliferation of breast cancer cell lines [54]. *FUT4* has been proposed as an effective diagnosis biomarker of breast cancer [55]. *PIGR* is known to be upregulated in breast cancer and other cancers [56].

Given the large number of associations,we run a gene set enrichment analysis on Reactome [57]. The top module was TP53 regulation of metabolic genes. It was not significant after correcting for multiple hypothesis.

If we consider only the very heterogeneous effects (estimated *r*<0.1), we find the following associations with *FDR*<0.1: *CMKLR1*, *CASC5*(*KNL1*), *SUSD1*, *RASSF4*, *AOC4P*, *ADHFE1*, *FAM71C*, and *RRN3P3*. *CASC5*(*KNL1*) is known as Cancer Susceptibility Candidate Gene 5 Protein. *RASSF4* is a member of the *RASSF* family of tumor suppressors. *AOC4P* is a lncRNA involved in hepatocellular carcinoma and colorectal cancer [58] and *ADHFE1* is a breast cancer oncogene [59].

The breast cancer metastasis data (GSE48091 [27]), measures the gene expression in primary breast cancer tissue in 166 cases where metastasis happened and 340 cases without metastasis. Only 2 genes reached the Bonferroni significance threshold for association with metastasis status: *TAOK1* and *BC042012*, and 2 more had low FDR: *RALB* and *CA428624* and they were only significant with our test (*FDR*=0.02). Both *TAOK1* and *RALB* were found to be underexpressed. *TAOK1* had a relatively low *p*-value by Limma (E-04) but the 3 other associations are specific to our test and correspond to low values of *r*. *TAOK1* was previously listed as a metastasis-associated genes in basal-like breast tumors [60] (through expression). *RALB*, also known as RAS Like Proto-Oncogene B, is known for its role in invasion and metastasis across cancers and specifically for breast cancer [61].

For both breast cancer datasets, we found interesting heterogeneous associations that would not be detected by tests looking for the broad differences between all cases and controls, illustrating the value in looking for aberration enrichment.

### Results across other types of -omics data

We ran our test on publicly available miRNA expression and DNA methylation datasets that we downloaded from GEO. Given the larger size of these datasets (thousands of samples in miRNA datasets and more than 450k features for methylation data), we used *k*=100 for the number of hidden confounders removed (using the PEER correction). Table 3 summarizes our findings with *k*=100. The results with *k*=30 are presented in the Additional file 1: Table S3.

**Table 3:** Experimental data findings: number of genes detected

Threshold	Bonferroni			FDR <0.1		
Method	Our test	Limma	∩	Our test	Limma	∩
RA methylation	119	2	1	506	3	3
Schizo. cortex	22	0	0	139	0	0
Schizo. blood	75	0	0	530	0	0
Breast miRNA	427	0	0	564	0	0
Ovarian miRNA	364	5	4	849	23	23

Our test returned a larger number of associations in these datasets, most of which were not discovered by Limma (see Table 3). In fact, using Limma or a *t*-test resulted in few to no associations, especially in methylation data. We did observe bimodality or mutimodality in the methylation levels of many sites and that can partially explain the poor performance of *t*-test and Limma. Non-parametric methods such as our test and Wilcoxon might be better suited for methylation data. Wilcoxon test returned a larger number of associations compared to Limma and *t*-test (see Additional file 1: Table S4). These associations were still fewer in numbers than our test and partially overlapped with our associations that corresponded to broad effects (large values of *r*).

In our analysis, given the large number of associations by our test, we prioritize those that affect a small proportion of cases (lower estimated values of *r*) rather than broad signals; these were also not found by Wilcoxon/Limma/*t*-test. Additionally, the large number of associations allows us to perform gene set enrichment analysis for the associated probes that are annotated to genes. Note that some of these associated probes are co-located and annotated with the same gene name. However, most of the genes we discuss below only have one associated probe.

In each dataset, to verify that our test is still well calibrated, we performed random label permutations and showed that we find no associations under the null.

#### DNA methylation in rheumatoid arthritis

The Rheumatoid Arthritis methylation data (GSE42861 [30]) measures DNA methylation for 354 RA patients and 335 controls. A large number of sites were found to be associated by our test. The top associations (FDR <10^−4^, *r*<30*%*) involve multiple sites near HLA genes such as *HLA-DQA1*, *HLA-DPB2*, *HLA-DRB1*, and *HLA-DMB*, along with other genes: *SLC43A2*, *MBD1/CXXC1*, *ALLC*, *LPP*, *ESYT2*, and *NMB*. Note that we also find other HLA genes such as *HLA-B*, *HLA-DRB5*, *HLA-DQB1*, and *HLA-DRB6* that are associated with *FDR*<0.01. *HLA-DRB1* is the strongest causal gene for RA [62]. In our data, 53 methylation sites were annotated to *HLA-DRB1*. Among these, 4 were found to be strongly associated by our test exclusively: cg04026937 (*FDR*=1.3*e*−03), cg06204447 (*FDR*=8.1*e*−05), cg18052547 (*FDR*=1.4*e*−02), and cg23899527 (*FDR*=2.2*e*−05). One (cg00598125) was found to have some association by Wilcoxon (*FDR*=0.06) and was sub-significant by our test (*FDR*=0.19) and did not correspond to an aberration enrichment pattern of association (*r*=0.6). We also looked at loci that are dysregulated in less than 10% of patients with FDR <0.1. Among the 12 loci we find *CHI3L1* (hypomethylated in *r*=9*%*) which is a rheumatoid arthritis autoantigen [63], SNW1 (hypomethylated in *r*=7*%*) which is a nuclear factor kappa B (NF) regulatory gene involved in RA pathogenesis [64] and *CAV1* (hypermethylated in *r*=9*%*) which is involved in NF-kappa-B activation in a T-cell receptor/CD3-dependent manner [65]. Among the genes mentioned above, *HLA-DQA1*, *HLA-DQB1*, *SLC43A2*, *MBD1/CXXC1*, and *NMB* and one site near each of *HLA-DRB1* are associated by Wilcoxon with FDR <0.1. These common associations always correspond to the higher values of *r* (*r*∈[0.26−0.3] when we selected only the hits with *r*≤0.3). All other associations are uniquely found by our test and many of which correspond to lower values of *r*≤0.2.

An enrichment analysis on Reactome [57] shows several immune system modules are enriched in the candidates returned by our test with *r*<0.3 and *FDR*<0.1. The module Class I MHC-mediated antigen processing and presentation is strongly enriched (*FDR*<8×10^−15^). The responsible genes are *CLCN7*, *TRIM41*, *ERICH1*, *HLA-B*, *TAP1*, *UBE2E2*, *UBAP2L*, *CBLB*, and *TAPBP*. The corresponding submodules: Endosomal/Vacuolar pathway, ER-Phagosome pathway and Antigen Presentation: Folding, assembly and peptide loading of class I MHC, are particularly enriched (*FDR*<8×10^−15^). The module Interferon Alpha/Beta signaling is also enriched because of genes *ZNF605*, *HLA-B*, *TAP1* (*FDR*<8×10^−15^).

While other methods are unable to recover the previous modules, the associations with Wilcoxon agree with our test on other modules containing *HLA-DRB5*, *HLA-DQA1*, *HLA-DRB1*, *HLA-DQB1* : TCA signaling, PD-1 signaling, Interferon Gamma signaling, MHC class II antigen presentation (the latter also containing *HLA-DMB*, *CAPZB*, *TAP1*, *HLA-DOB*). This is not surprising since MHC class II antigen presentation is very well known to be involved in RA [66]. Finally, there are several modules related to NOTCH signaling which is also known to play a role in RA [67] (containing the following associated genes *NCOR2*, *HDAC4*, *HDAC2*, *SNW1*, *ERICH1*, *FBXW7*, *MIB2*, *PSEN2*, *RBPJ*, *NOTCH4*). Overall, our test detected several associations with potentially disease-relevant genes and pathways, some of which were not detected using any other approach.

#### DNA methylation in schizophrenia

The schizophrenia methylation datasets (GSE74193 [31] and GSE80417 [32]) respectively describe the DNA methylation in dorsolateral prefrontal cortex and whole blood. After preprocessing (Additional file 1: Supplementary methods), the first dataset had 191 schizophrenia cases and 335 controls and the second dataset had 305 cases and 333 controls.

In the brain, the top loci with proportion of affected cases *r*<30*%* are by the genes *HLA-DRB6*, *SOX2OT/SOX2*, intergenic region at loci cg23330385, *NAALADL2*, *LIN7A*, *SOAT1*, *HLA-DRB1*, *ALDH3B2*, *LOC81691*, *NMNAT2*, *CNRIP1*, *TTC23L,* and *SLC16A12* (all associations are with FDR <0.01).

At least 4 of these genes have been implicated with schizophrenia in the literature. For example, *LIN7A* is at the overlap of several rare CNVs associated with schizophrenia in [68] and induced overexpression of *CNRIP1* is known to cause a schizophrenia like-phenotype in mice [69]. *NMNAT2* is important for the maintenance of neurons and is known to be neuroprotective in several models of neurological disorders [70], while *HLA-DRB1* is the most frequently reported genetic association to schizophrenia [71]. Furthermore, looking at the sites with lower proportion of affected samples (*r*<10*%*), we find the 6 associated sites with FDR <0.1: *CYFIP1*, *ST6GALNAC1*, *ABCA8*, *CPSF6*, *C6orf25,* and intergenic site cg25008182. The site near the *ST6GALNAC1* gene is hypomethylated in 7% of the cases, and is known to be associated (through hypomethylation) with schizophrenia and bipolar disorder in an identical twin methylation study who are discordant for these diseases [72]. *CYFIP1*, here hypermethylated in 8% of the cases, was previously associated with schizophrenia and autism through CNVs and is known to regulate the balance between synaptic excitation and inhibition [73]. The *ABCA8* gene is important for lipid metabolism in oligodendrocytes, myelin formation and maintenance, and *ABCA13* from the same subfamily is associated to schizophrenia through GWAS [74]. The genes listed above are uniquely found by our test except for *NMNAT2* which was found with FDR =0.08 by Wilcoxon and FDR =0.002 by our test. This shows that looking for aberration enrichment in addition to traditional approaches can lead to novel associations that might improve our understanding of disease.

The Reactome gene set analysis found an overlapping group of gene sets previously found by Wilcoxon and our test of rheumatoid arthritis, such modules containing the genes *HLA-DRB5*, *HLA-DQA2*, *HLA-DRB1* : TCA signaling, PD-1 signaling, Interferon Gamma signaling, MHC class II antigen presentation (the latter also containing *RACGAP1* and *ITFG1*). In schizophrenia, these modules are only detected through our test and not through Wilcoxon. This result is not surprising and is consistent with the strong associations between the HLA locus and schizophrenia found in different studies [75]. We also report the following modules of unknown relevance to schizophrenia: Glucuronidation with FDR= 8.81*E*−04 (genes *UGT1A3* to *UGT1A10*) and Phase II - Conjugation of compounds with FDR= 5*E*−03 (*SLC35B3*, *GGT7*, *MGST3*, and *UGT1A3* to *UGT1A10*).

In blood, the results were less interesting with a very large number of associated sites in Table 3 (Wilcoxon also found 61 and 242 sites by Bonferroni and FDR respectively) and less obvious associations with schizophrenia in previous literature among our immediate top genes. The top 10 associations with *r*<0.1 that are close to genes are near *AP2S1*, *MYH7*, *DSCR3*, *C14orf182*, *TMCO1*, *PRR25*, *LOC389333*, *SELS*, *XKR6*, and *DGKZ*. All of these associations have FDR <0.05. *DSCR3* (Down Syndrome Critical Region Gene 3) has previously been associated with neuroticism in a genome wide linkage study [76]. *C14orf182* has been associated with schizophrenia in a whole genome sequencing study done in discordant twins [77]. *DGKZ* is located within a schizophrenia GWAS loci and is further known to be dysregulated in schizophrenia patients [78, 79].

Both in brain and in blood, our test is recovering novel associations with genes/loci potentially relevant to schizophrenia which would not be picked by other methods because of the heterogeneous nature of these associations (aberration enrichment).

#### miRNA in breast and ovarian cancer

The breast cancer miRNA data (GSE73002 [28]) describes the serum miRNA levels of 1280 breast cancer cases and 2686 non-cancer controls. The ovarian cancer miRNA data (GSE106817 [29]) describes the serum miRNA levels of 399 ovarian cancer cases and 3647 non-ovarian cancer controls (includes 859 samples from other cancers). After preprocessing (see Additional file 1: Supplementary methods), we ran Limma and our test on both datasets. Overall 963 and 2565 miRNAs measurements were made in the breast cancer and ovarian cancer dataset respectively. Out of those measured miRNAs, a relatively large proportion was found to be associated to the cancer status according to our test as shown in Table 3. We attempted to use larger values for the number of PEER factors *k* but this did not substantially reduce the number of associated hits (Additional file 1: Table S2). For example, in the breast cancer dataset, our test uncovered 483 associated miRNAs for *k*=30. Using *k*=100 or *k*=200 only reduced that number to 427 and 425 respectively. Similarly in the ovarian cancer dataset, 462 associations were detected by our test for *k*=30 and that number reduced to 364 and 352 for *k*=100 and *k*=200 respectively. Using Limma or a *t*-test returned very few to no associations while Wilcoxon returned a smaller number of associations than our test.

To show that these associations are not artifacts from our test, we performed random permutations of the labels and found zero associations, meaning that there does not seem to be an inflation for type 1 errors for our test.

One possible explanation of these results is that cancer generates a large number of effects that are not homogeneous across patients. This is a well-known phenomenon [80]. Heterogeneous downstream effects of cancer might include events such as large copy number changes, structural variants, large effects on chromatin conformation and epigenetics. Any of these events can result in dysregulation of miRNAs and any single event could be happening in a smaller proportion of cancer cases. The heterogeneity of cancer presentations across patients could also lead to a heterogeneity of downstream effects that would be observed as a large number of associations by our test. This result is consistent with the large number of associations we also observed in gene expression data in the breast cancer dataset compared to non-cancerous diseases. In the “Breast cancer” section, we observed 453 genes with FDR <0.1 and 1506 genes with FDR <0.2 in association with breast cancer.

Under this assumption of numerous heterogeneous downstream effects, it is difficult to pinpoint miRNA dysregulations that would be drivers of cancer among a very large number of associations. This is particularly problematic when we have a high proportion of associated features among all features (>20*%* of miRNAs have *FDR*<0.1 in our data). This shows the limitations of directly applying our test to cancer, where there is an accumulation of heterogeneous passenger events.

However, we argue that our test can be used in this context, but not for the task of feature selection (identifying relevant cancer miRNAs). Instead, we use it for identifying features (in this case, miRNAs) that are helpful for classifying individuals into cases vs controls. The argument here is that even if (most of) the associations are just downstream heterogeneous effects, they can still be used as biomarkers of cancer.

For each dataset, we split the data into a discovery cohort and a held-out cohort (not to be used for feature selection or training). We run our test on the discovery data to uncover miRNAs with heterogeneous associations. Many of our associations are found with *r*<30*%*, meaning the considered miRNA’s association is produced by only a proportion of individuals with extreme values (overexpressed or underexpressed). We define the intervals of expression that are responsible for the association (using the index at which the standardized enrichment score is maximal; see Fig. 1 as an example), then we assign a value of zero to all other individuals that are not in the interval of interest. This manually introduced non-linearity helps the model focus on meaningful dysregulations rather than considering the full expression spectrum as a whole for each miRNA. We use a lasso-penalized logistic regression classifier (R package glmnet [81]). More details about this experiment can be found in Additional file 1: Supplementary methods.

We report our results in Table 4 where we used a combination of feature selection and a logistic regression classifier to differentiate cancer patients and healthy controls. We select either the top 300 features by Limma or the top 300 heterogeneous features (*r*<0.3) by our test. We optionally transform the top features of our test by assigning a value of zero to all individuals outside of the interval of expression that drove the association (Top Hetero. transformed column). We also compare to using all features or only the features previously used in the literature for this classification problem (a set of 5 and 10 miRNAs respectively for the breast cancer data and the ovarian cancer data).

**Table 4:** Cancer-control classification performance on held-out data with area under the precision-recall curve (AUPRC) after feature selection by different methods

Features	Top 300 Limma	Top 300 heterogeneous	Top Het. features	All features	Literature
	features	features	transformed		features
Ovarian cancer	0.695	0.743	0.948	0.696	0.503
Breast cancer	0.530	0.612	0.965	0.632	0.541

Using the features selected by our test leads to a much better classification performance compared to when we use the top features returned by Limma or when we use all features in the classifier. Our approach reaches AUC and AUPR over 0.94 for both datasets which is a much better performance than other feature selection approaches such as using Limma. The transformation of keeping only the expression values within the aberrant interval defined by our test is very helpful. This is consistent with our previous observation in simulation experiments that a logistic regression is not good at handling/detecting features with heterogeneous effects (see the “Simulating gene expression data” section). The non-linear data transformation based on our test results seems to address this limitation of logistic regression. In fact, using a non-linear classifier such as random forest (R package RandomForest [82]) leads to a very similar performance to using transformed features in the logistic regression case, but our approach is easier to interpret. The Random Forest AUPR is 0.92 and 0.98 on the held-out data respectively for the ovarian cancer and the breast cancer datasets (see Table 4). However, the random forest performance is unchanged (±0.01 AUPR) whether we use Limma’s top features or those of our test and whether we transform the features or not.

It is very important to note that the problem of classifying cancer cases from controls has already been solved with very high accuracy for the same miRNA datasets [28, 29]. A full classification performance is achievable even with few features because of the very broad difference observed between data originating from cases and controls. For example, running Limma, Wilcoxon or our test on the non-preprocessed data results in almost all miRNAs being strongly differentially expressed. In this proof of concept, we used processed data where 100 hidden PEER factors were removed. Some of these factors correspond to broad signal of cancer that could easily separate cases and controls. In fact, we verify that using 20 of those hidden factors as features, we can recover a perfect classification with logistic regression or random forest. By removing the 100 hidden factors from the data, we made the classification problem harder than the one previously solved on the original data. In this proof of principle, our goal is to prove that heterogeneous disease signals do exist and that they have predictive value beyond broad signal. Using our test to detect and process these heterogeneous signals, we showed that we can improve upon the performance of a linear classifier in an interpretable way.

## Discussion

In this paper, we presented a statistical test for detecting a pattern of association different from an overall shift in mean or variance between cases and controls. We call this pattern “aberration enrichment” or association with “heterogeneous effects”. Our test works in a case/control setting with a continuous input variable (such as a gene’s expression) and scales to hundreds of thousands of variables.

Through the use of simulations, we showed that our test is more adapted at uncovering associations with heterogeneous effects compared to the widely used statistical methods. Our test is well calibrated and uses permutations to assess the significance of the results. The power of our test is inferior or on par to traditional approaches in the classical setting, i.e., for detecting broad signal with no heterogeneous effects, but becomes vastly superior when the signal of interest concerns a smaller proportion of the cases (*r*≤30*%*).

By applying our test to complex diseases and several experimental gene expression datasets, we showcase its ability to detect novel potentially disease-relevant genes that would not be detected by traditional differential expression methods. We further applied our test to other omics data types (miRNA and methylation) and reported novel associations.

Many of the genes found by our test do not exhibit a broad signal across the disease cases. This makes their association with the disease less likely to be a homogeneous downstream consequence of the disease itself. However, that does not imply these genes are causal for the disease. It is still possible that some confounding variables (such as the environment or a drug) is affecting a subset of the cases. It is also possible that the considered disease is heterogeneous enough to generate a multitude of heterogeneous downstream effects on the measurements that are unobserved in the controls. For example, cancer may generate heterogeneous downstream consequences such as large CNVs and chromosomal rearrangements which would appear to our test as consistent outliers enriched in cancer cases but not in controls. Our test cannot distinguish causal factors from heterogeneous downstream consequences. Similarly to the widely used differential expression approaches, our test can return a very large number of associations in some contexts, thus rendering a downstream search of causal elements very difficult.

In real/experimental data, it is important to correct for known and hidden confounders in order to remove broad irrelevant signals and obtain a small set of associated genes. Here we used PEER [33] to correct for confounders. It is always possible that some complex/non-linear hidden confounders or other broad effects (such as cell-type proportion heterogeneity across patients) are not being fully removed by PEER. It is also possible that this procedure of removing hidden confounders might also be removing signals that are relevant to the causal mechanisms of the disease in our experiments (leading to false negative genes). Furthermore, the procedure of correcting for confounders generally works under the assumption that the confounders affect the mean, but some confounders could be affecting the variance of the measurement of interest [83]. If that is the case, it is possible to identify false associations driven by confounders that have different variances in cases and controls. Better upstream procedures for correction which consider the effect of confounders on variance will be beneficial for all the methods considered but especially so for methods that look for beyond the effect on the mean.

Currently, the need for permutations makes the method slower than the widely used statistical tests. Especially if we want to accurately measure very low *p* values. More work needs to be done to better model our test statistic (the max over correlated standardized enrichment score variables) in order to obtain a closed form solution. Currently the null distribution over test statistics is not analytically computed and it does not clearly fit any known parametric distribution we tried. (The max over dependant standardized weighted hypergeometric variables is not easy to model. A polynomial approximation works to fit the tail but it is hard to justify so we did not rely on it.) In terms of running time, our test in its current form can still be easily applied on a personal laptop and it takes 15 min to run on a full gene expression dataset with sample sizes under 500. It can take around 6 h on a full DNA methylation dataset with up to 450,000 features.

The statistical test presented in this paper could be applied to other datasets and other fields beyond complex diseases and omics data. Wherever a 2-group test is used (such as Wilcoxon, t-test, or the equivalent logistic regression), our test could be a complimentary analysis, especially where we might expect a non-homogeneous difference between the groups. For example, in randomized clinical trials, we often compare a continuous measure of response (a change from baseline in a measure of disease severity) between individuals who took the drug and individuals who took a placebo in order to prove the drug’s efficacy. In a heterogeneous treatment effect (HTE) setting where the drug has a clear positive effect on only a proportion of patients, i.e., responders, traditional tests might be underpowered to detect the efficacy by testing for the difference in mean between the two groups. Our test could greatly benefit clinical trials because of the gain in power for detecting the drug’s heterogeneous effect.

As a downstream analysis after a heterogeneous association is detected by our test, one can pinpoint the group of individuals with the aberration enrichment pattern and attempt to characterize that group by finding commonalities. Such an approach would be more focused than unsupervised clustering methods, because it zooms in on only the group that led to a statistically significant association using the case-controls labels.

## Conclusions

We present a novel statistical test that is particularly suited for the detection of heterogeneous associations. Our test showed vastly better performance on simulations compared to existing approaches including other widely used statistical tests. We showed the usefulness of our test on experimental data analyses by applying it to different genomics data types and recovering interesting disease associations. Beyond our current results, our test can be widely applicable to a large number of problems where heterogeneous effects are suspected, including clinical trials data with heterogeneous treatment effects.

## Supplementary Information

**Additional file 1** Supplementary methods, Figures and Tables.

## Data Availability

The data that support the findings of this paper are publicly available on GEO. This includes the following datasets: Alzheimer disease data GSE63063 [23], Parkinson disease data GSE99039 [24], inflammatory bowel disease data GSE73094 [25], heritable breast cancer data GSE47862 [26], breast cancer metastasis data GSE48091 [27], breast cancer miRNA data GSE73002 [28], ovarian cancer miRNA data GSE106817 [29]), rheumatoid arthritis methylation data GSE42861 [30], and schizophrenia data GSE74193 [31] and GSE80417 [32]). The package for our statistical test has been published on the Comprehensive R Archive Network (CRAN) as “aziztest”: https://cran.r-project.org/web/packages/aziztest/index.html. It can be installed using: install.packages(“aziztest”) The code [20] used in this paper (simulations, preprocessing, visualisation, etc.) is available on: https://github.com/azizmezlini/Aberration_Enrichment_Code.
