# Supplementary material for: Finding associations in a heterogeneous setting: statistical test for aberration enrichment
Source: Genome Med. 2021 Apr 23;13:68. doi: 10.1186/s13073-021-00864-4 (PMC8066476; doi:10.1186/s13073-021-00864-4)
Supplement: Supplementary file 1 — Additional file 1 Supplementary methods, Figures and Tables. [file 13073_2021_864_MOESM1_ESM.pdf]

Finding associations in a heterogeneous setting: Statistical test for aberration enrichment

Aziz M. Mezlini, Sudeshna Das and Anna Goldenberg

Supplementary Methods

Statistical test for aberration enrichment

To calculate the enrichment score for a given variable of interest, we first sort the samples by that variable, then we walk through the list taking positive steps when we encounter a case and negative steps when we encounter controls. The enrichment score at a given position in the ranked list of samples, is a weighted sum of increments (for cases) and decrements (for controls): The formula for the kth position of the enrichment score S_k is:

$$\begin{aligned} S_k &= \frac{1}{n_1}\sum_{i=1}^{k} w_i X_i - \frac{1}{n_0}\sum_{i=1}^{k} w_i(1 - X_i) \\ &= (\frac{1}{n_0} + \frac{1}{n_1})\sum_{i=1}^{k} w_i X_i - \frac{1}{n_0}\sum_{i=1}^{k} w_i, \end{aligned}$$

where n_1 and n_0 are the total numbers of cases and controls, $\mathbf{w}$ is the vector of weights, and X is the indicator variable describing which individuals are cases (1) and which are controls (0). The weights w can be the absolute values of the standardized levels of the variable of interest, thereby heavily weighting aberrations of large magnitude by making them correspond to larger steps in the walk. The standardization used in this paper is a subtraction of the sample mean and a division by the standard deviation. In practice we set the minimum on w to be 0.5 so that even individuals that are not outliers (near 0 standardized expression) can still contribute to our test. We chose to use this weighting scheme across all our experiments in order to make our test sensitive to the magnitude of aberration while still considering the ordering across all individuals when looking for an enrichment in cases versus controls. If we take $w = 1$ a constant across individuals, then only the ordering matters (similarly to Wilcoxon) and the scale no longer have an impact on the test statistic. This change of weighting scheme has a mild effect on the associations discovered in practice (See Additional file 1: Figure S9).

Under the null hypothesis, X is a random permutation of the case/control labels. The total number of cases and controls being fixed, each X_i follows a Bernoulli distribution with a probability $\frac{n_1}{n_1+n_0}$. The expectation and variance for the enrichment score at position k are:

$$
\begin{aligned}
E_{null}[S_k] &= (\frac{1}{n_0} + \frac{1}{n_1}) \sum_{i=1}^{k} w_i E[X_i] - \frac{1}{n_0} \sum_{i=1}^{k} w_i \\
&= ((\frac{1}{n_0} + \frac{1}{n_1}) \frac{n_1}{n_1 + n_0} - \frac{1}{n_0}) \sum_{i=1}^{k} w_i = 0 \\
Var_{null}(S_k) &= (\frac{1}{n_0} + \frac{1}{n_1})^2 Var(\sum_{i=1}^{k} w_i X_i) \\
&= (\frac{1}{n_0} + \frac{1}{n_1})^2 \mathbf{w_{1:k}^t} \Sigma_{1:k} \mathbf{w_{1:k}} \\
&= (\frac{1}{n_0} + \frac{1}{n_1})^2 [\frac{n_1 n_0}{(n_1 + n_0)^2} \sum_{i=1}^{k} {w_i}^2 - \frac{n_1 n_0}{(n_1 + n_0)^2 (n_1 + n_0 - 1)} \sum_{i=1}^{k} \sum_{j \neq i}^{k} w_i w_j] \\
&= (\frac{1}{n_0} + \frac{1}{n_1})^2 \frac{n_1 n_0}{(n_1 + n_0)^2} [\frac{n_1 + n_0}{n_1 + n_0 - 1} \sum_{i=1}^{k} {w_i}^2 - \frac{(\sum_{i=1}^{k} w_i)^2}{(n_1 + n_0 - 1)}] \\
&= \frac{1}{n_0 \cdot n_1} \frac{n_1 + n_0}{n_1 + n_0 - 1} (\sum_{i=1}^{k} {w_i}^2 - \frac{(\sum_{i}^{k} w_i)^2}{n_1 + n_0}),
\end{aligned}
$$

where $\Sigma_{1:k}$ is the covariance matrix of $X_{1:k}$, which has value $C_0 = \frac{n_1 n_0}{(n_1+n_0)^2}$ on the diagonals and $C_1 = -\frac{n_1 n_0}{(n_1+n_0)^2 (n_1+n_0-1)}$ off the diagonal.

We standardize the enrichment scores by dividing them with the square root of the variance under the null $Var(S_k)$. All positions being comparable after standardization, we select the max standardized score across all positions as our test statistic $S_{max} = max_{k \in [1, n_0+n_1]} (S_k / \sqrt{Var_{null}(S_k)})$.

The whole procedure described above is performed in both directions: sorting patients by increasing or decreasing expression levels and computing the max standardized enrichment score twice. Only the higher max standardized enrichment score is taken. In the example of gene expression data, this allows us to properly test for enrichment in overexpression and underexpression simultaneously. In a setting where only one direction of effect ought to be tested, we could perform the procedure on only one direction for a small gain in power.

We assess significance by running permutations on the case/control labels and observing how often we obtain a higher value for S_{max}.

If we have an association, we can look back at which S_k corresponds to S_{max}. This will define the interval of values considered to be associated with cases versus controls. From there we can compute several useful interpretable quantities: such as the number of cases and controls in that interval, the odds ratio of being in that interval for cases, and the estimated r which is the proportion of cases affected (cases in that interval divided by all cases). We can also identify which individuals are affected and further study that group or enrich for it in future experiments. For example, in a clinical trial setting, our test would not only detect the heterogeneous drug effect but also return the identity of the patients who were affected (responders) which can be further analyzed and characterized.

Experimental data and preprocessing

The data downloaded from GEO is already in Log scale, we standardize it, apply PEER [1] to remove known confounders (if provided) and 30 hidden factors. Then we test the residuals for differential expression analysis. Genes with missing values and patients missing key clinical variables were removed from the analysis.

Our focus here is to find genes that follow the pattern of aberrant differential expression. Therefore we want to remove any broad signal in the data caused by confounders or hidden variables

or the consequences of the disease itself (compensatory pathways, drug effects, etc). PEER helps us remove such broad signals. The same approach was used in previous work in order to detect expression outliers associated with rare eQTLs [2]. Note that PEER can potentially reduce the number of differentially expressed genes if some of the inferred PEER factors corresponds to broad effects of the disease status. Given that the hidden PEER factor can model pathways and transcription factors, it is also possible that some truly differentially or aberrantly expressed genes signal will be removed by PEER if these genes are regulating a large number of other genes' expression. However, attempting the same analysis without removing any hidden factors resulted in a too large number of associations: in some datasets, almost all genes were significantly associated by any method (Limma, Wilcoxon, our test). This would make futile any attempt at recovering relevant mechanisms of disease. Therefore, we decided to go with the PEER hidden factor removal for the datasets analysed in this paper.

The Alzheimer data (GSE63063 [3]) contains the gene expression of 284 AD patients, 189 MCI (mild cognitive impairment) and 238 healthy matched controls measured in blood. It comes in two batches (USA and UK) using the Illumina HumanHT-12 V3.0 expression beadchip and Illumina HumanHT-12 V4.0 expression beadchip platforms respectively. We correct for gender, age, ethnicity, batch/platform in addition to the 30 PEER hidden factors.

The Parkinson data (GSE99039 [4]) contains the whole blood gene expression of 205 IPD cases and 233 controls, we corrected for gender and batch in addition to the 30 PEER hidden factors.

The inflammatory bowel disease data (GSE73094 [5]), contains the colon and terminal ileum gene expression of 608 samples from CD patients, 331 from UC patients and 50 samples from non IBD individuals. Overall 374 samples were taken during inflammation and 609 taken from non inflamed tissues. When we looked for association with CD versus UC and vice-versa, we used the non-inflamed samples and we selected individuals with one non-inflamed sample (a few individuals had multiple samples taken). This resulted in 181 UC samples and 314 CD samples. We corrected for group (code IBD2, IBD3, IBD4), tissue of origin, and 30 PEER hidden factors. When we looked for association to inflammation status, we took all samples (374 inflamed and 609 non inflamed) and we corrected for group, disease type and tissue in addition to the 30 PEER hidden factors.

The heritable breast cancer data (GSE47862 [6]) measures the gene expression in peripheral blood in 158 women with breast cancer and 163 controls. 226 women have a family history of breast cancer, 93 of which carry BRCA mutations. We corrected for cohort (Ontario or Utah) and 30 PEER hidden factors.

The breast cancer metastasis data (GSE48091 [7]), measures the gene expression in primary breast cancer tissue in 166 cases where metastasis happened and 340 cases without metastasis. We corrected for training/validation status and 30 PEER hidden factors.

The first schizophrenia methylation data (GSE74193 [8]) describes the DNA methylation in dorsolateral prefrontal cortex for 191 cases and 335 controls (after QC, removing duplicates and removing 8 cases whose reported gender differed from the predicted gender). We corrected for gender, race, batch, tissue composition in addition to the 100 PEER hidden factors.

The schizophrenia methylation data (GSE80417 [9]) measures the whole blood DNA methylation for 305 cases and 333 controls. We corrected for gender and age in addition to the 100 PEER hidden factors.

The Rheumatoid Arthritis methylation data (GSE42861 [10]) measures DNA methylation in peripheral blood leukocytes for 354 RA patients and 335 controls. We corrected for gender, age and smoking status in addition to the 100 PEER hidden factors.

The breast cancer miRNA data (GSE73002 [11]) describes the serum miRNA levels of 1280 breast cancer cases and 2686 controls. The ovarian cancer miRNA data (GSE106817 [12]) describes the serum miRNA levels of 399 ovarian cancer cases (including 79 borderline ovarian tumor) and 3647 non-ovarian cancer controls. The controls included 2759 healthy individuals and 859 individuals with other solid cancers (not ovarian). There were no additional clinical variables to use as confounders (In the ovarian cancer dataset, age had a missing value in the controls). In the classification experiment, we selected only the samples labeled ovarian cancer (320) and healthy controls (2759) for the ovarian cancer data. We corrected for 100 PEER hidden factors.

Permutations analysis

To assess the significance of a considered variable's max enrichment score, we run permutations on the case/control labels and we calculate how often the max standardized enrichment score generated by a permutation exceeds the score obtained on the real example. In order to compute accurate p-values, we need to run a very large number of permutations which can be computationally prohibitive. To reduce the number of unnecessary permutations we tried two approaches and found they lead to the same result.

Approach 1: For variables(e.g. genes) that are clearly not going to be significant, we do not need a high level of accuracy in estimating the p-value. Therefore, we adopt a gradual approach of successively running 100,1000,10000,100000, 1000000,10000000,100000000 permutations only moving forward to the next step if less than 10 trials yielded higher test statistics than the real test statistic. We used this approach for all experimental gene expression datasets. By repeating the full experiment with more permutations every time, we essentially end up wasting a fraction of computations (the permutations from each previous cycle) but that is negligible compared to the gains in the majority of the genes where high accuracy is not needed to determine the clear absence of association. The factor 10 and the condition less than 10 trials work really well in practice, as we observed no bias in p-values estimations compared to the full permutation p-values.

Approach 2: When there is a large number of highly associated variables in a dataset, computing permutations for each variable can still be very slow using the first approach. By plotting the test statistic and the log of the permutation p-values for all considered variables on the same graph, we observed that the function mapping test statistic and p-value is clearly monotonous and that the p-values could easily be predicted from the test statistic alone independently of the gene considered (See Additional file 1: Figure S5). This can be explained by the fact that the enrichment scores were standardized by accounting for each position and set of weights w, generating test statistics values (max over standardized enrichment scores) that are therefore comparable quantities across the genes/factors tested in the same dataset.

Under this assumption, there is no need to compute permutations separately for every gene/factor. Instead, we can keep the case/control labels and compute permutations on only one variable or even on a vector sampled from a Gaussian and then use those permutations' test statistics to compute all the p-values from the test statistics (proportion of permutation test statistics that are higher than the true test statistic). We easily verify the quality of this approach by plotting the permutation p-values computed on only one Gaussian vector versus the p-values computed by doing permutations separately on every gene in Additional file 1: Fig S5. The correlation between the log p-values was 0.988. For datasets such as methylation data where there is a very large number of features, it is computationally prohibitive to compute permutations on every feature, especially given that a large number of permutations (10^8) is needed for every feature to reach a useful level of accuracy for p-values (multiple hypothesis burden). The second approach was particularly useful in these datasets where approach 1 or full permutations were prohibitively slow. The running time on a personal laptop was 15 minutes and 4 seconds for a gene expression dataset (Alzheimer Disease) and around 6 hours for a DNA methylation dataset (Schizophrenia brain).

Note that we do not assume that the mapping from test statistic to p-values is universal across datasets. Our second approach is applied separately on every dataset. The sample sizes and proportions of cases and controls are constant characteristics across phenotype permutations and could influence the mapping from the max standardized enrichment scores to p-values.

In our real data experiments, for the very significant genes/ methylation sites/ miRNAs with computed permutation p-value less than 1 over number of permutations (10000000 permutations or 100000000 for methylation data), we estimated the plotted p-value by fitting a linear regression of log(p-value) as a function of a third order polynomial of the test statistic. We are interested in modeling the relation between the higher test statistics and the corresponding p-values. Therefore the regression model was fitted on the random permutations that reached a p-value of 0.05 or under among the set of all permutations performed. We verified that this procedure can lead to an accurate estimation of the low p-values, but in this paper we only used it for visualization

purposes when plotting the p-values below the inverse number of permutations in Figure 7-A. The significance of all novel associations reported was re-verified and was not affected by this approximation procedure since we only apply it on the very significant associations ($p < 10^{-7}$, or $p < 10^{-8}$ for methylation data). Note that when a p-value is very significant (less than the inverse of the number of permutations), our implementation returns the inverse of 1 plus the number of permutations by default as suggested in [13]

Classification of cancer versus healthy from miRNA data

We held-out an equal number of cases and controls from the data corresponding to 15% of the number of cases. We performed a cross validation on the remaining individuals. For each fold, we use our statistical test on the training data to perform feature selections then feed the selected and transformed features to an L1-logistic regression classifier (glmnet R package). We used the area under the Precision-Recall curve (AUPRC) to evaluate the performance. The hyperparameters of the classifier (here the regularisation λ) are chosen on the validation set and we report the performance of the model on the held-out test data (never seen by our test and by the classifier).

For feature selection, we use the same number of features for Limma and our method (300). For our method, we select only the heterogeneous features (aberration enriched) with $r < 0.3$. The optional feature transformation is based on the results of our test. For every feature, we determine the interval of aberrant expression driving the association. When the association is due to under-expression, this corresponds to all levels of expression lower than the expression level of the individual for which the max standardized enrichment score was reached. When the association is due to over-expression, this corresponds to all levels of expression higher than the level of the individual for which the max standardized enrichment score was reached (See Figure 1 for an illustration of this interval of interest). Once the interval of interest is defined, the feature transformation consists in assigning a value of zero to any individual outside that interval.

Supplementary Tables and Figures

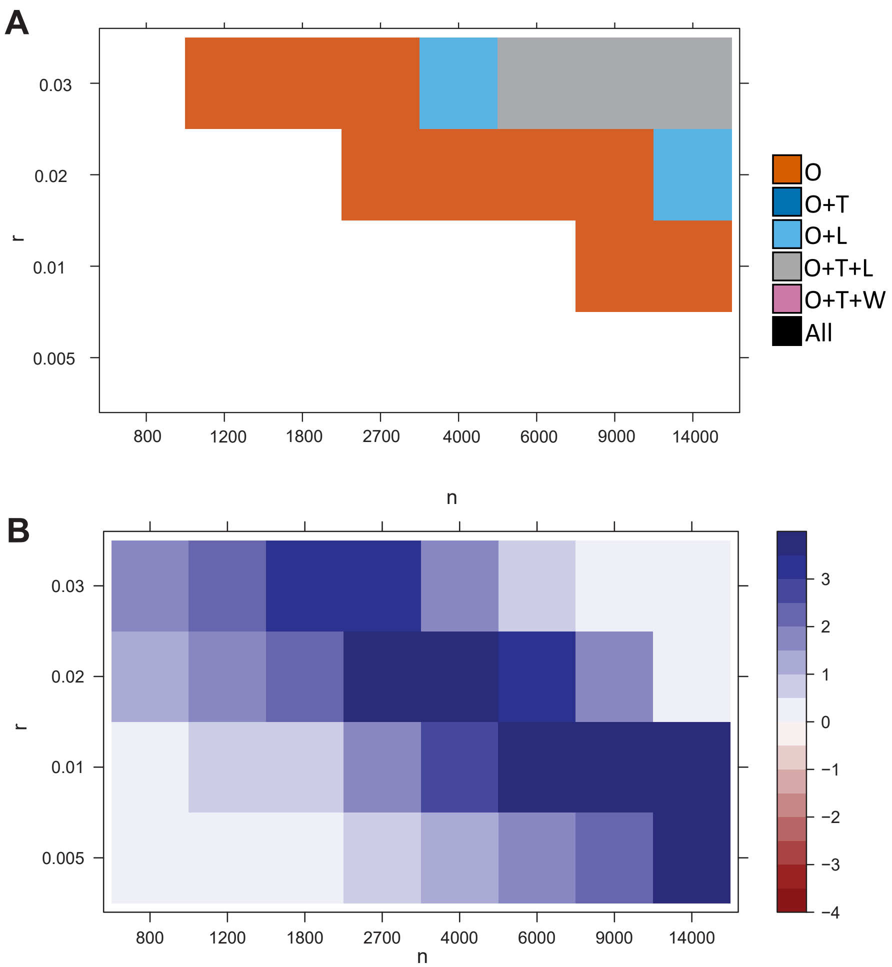

Figure S1: A Ability of the different tests to detect the association for very low values of r. A method is able to detect the signal if the p-value is lower than the threshold in the the majority of 200 reruns. Here we show the results for $d = 3$. **B** Comparison of the p-value magnitude between our aberration enrichment test and the best out of t-test, Levene and Wilcoxon test. depending on simulations parameters n (sample size) and r (proportion of affected cases). Here we show the results for $d = 3$. The colors indicate the difference in log10 between the p-values returned. For example 2 indicates that our test's p-value is two orders of magnitude (100 times) smaller than that of the best other method. We capped the maximal difference at 4 for visual clarity. In reality the average difference in magnitude between our p-values and those of other methods can be much larger than 4 order of magnitude (10000 times).

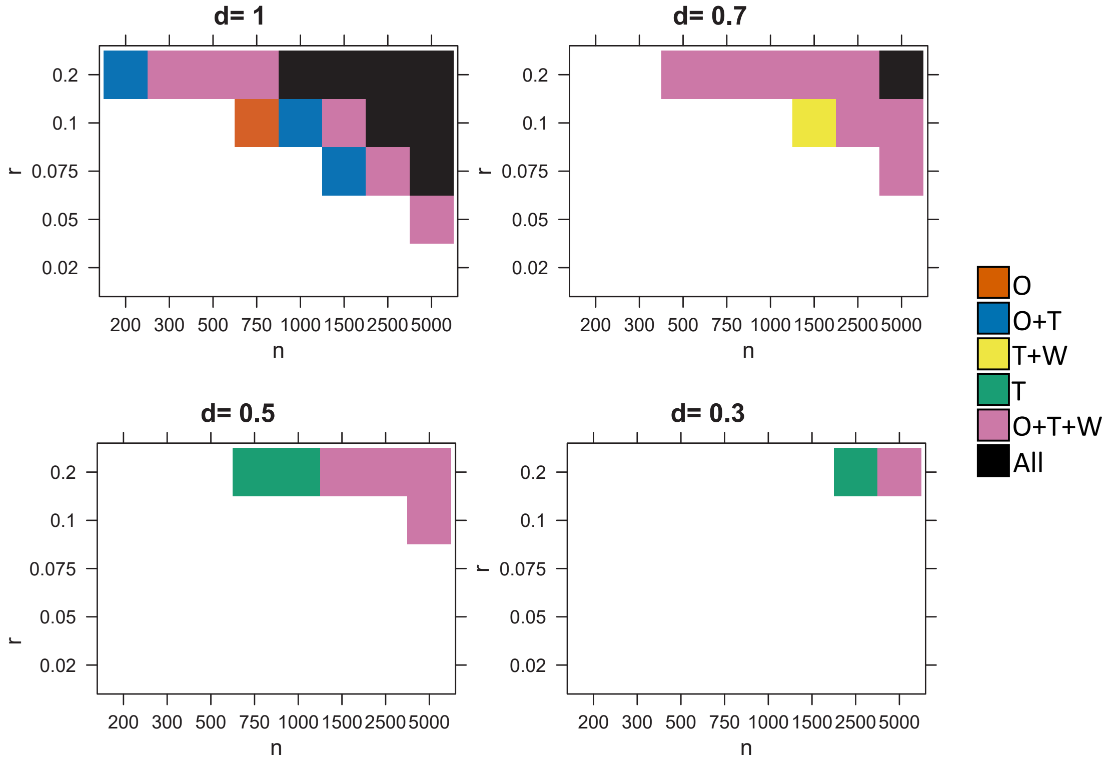

Figure S2: Ability of the different tests to detect the association for low perturbation magnitudes d, depending on simulations parameters n (sample size) and r (proportion of affected cases). A method is able to detect the signal if the p-value is lower than the threshold in the the majority of 200 reruns.

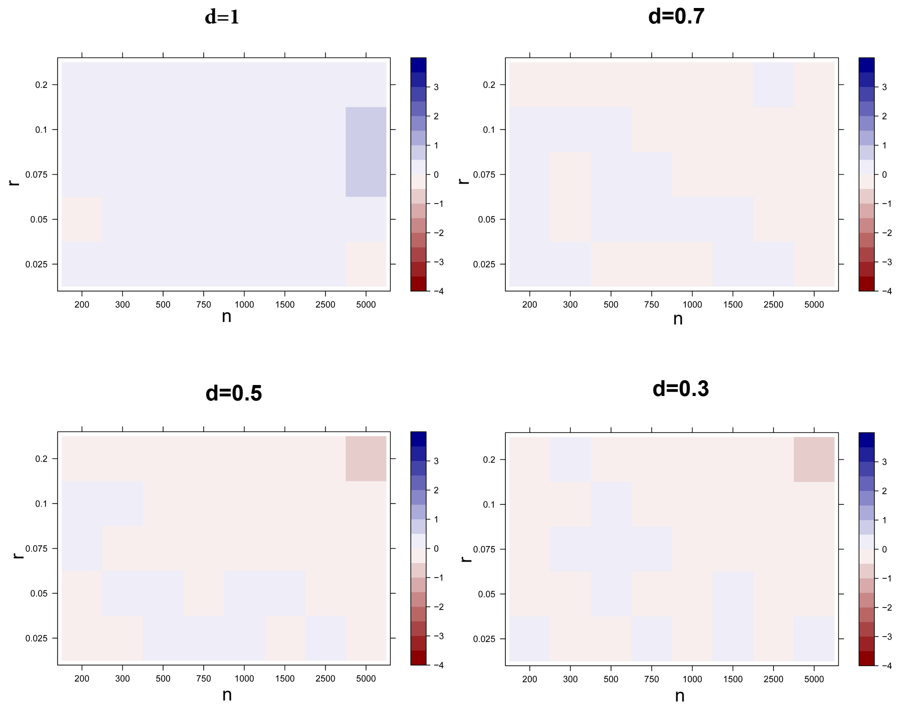

Figure S3: Comparison of the p-value magnitude between our aberration enrichment test and the best out of all other methods for smaller sizes of perturbation d, depending on simulations parameters n (sample size) and r (proportion of affected cases). The colors indicate the average difference in log10 between the p-values returned by both method. 200 reruns were performed. Blue is for when our method is better than the best of the other methods (t-test, Wilcoxon and Levene)

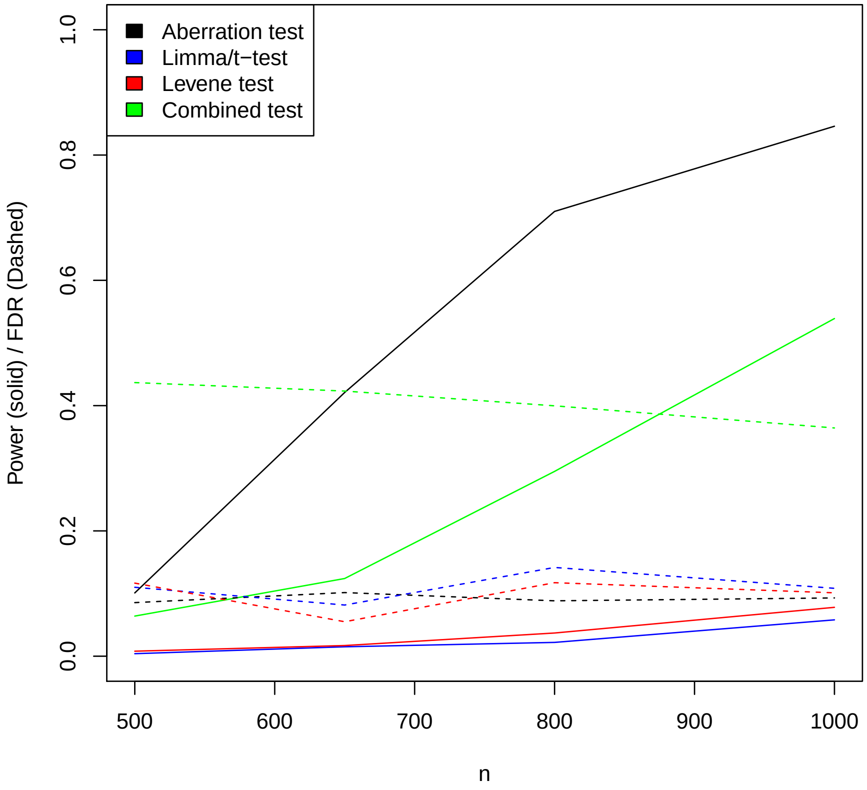

Figure S4: False discovery rates (hashed) and power (solid) in the harder setting where $r = 0.05$ (proportion of affected cases) with larger sample sizes n. Ability of the different tests to detect the 10 simulated true genes among 25000. We fixed $d = 3$. We used an FDR threshold of 0.1 for all methods. The average performance over 100 simulations is shown here.

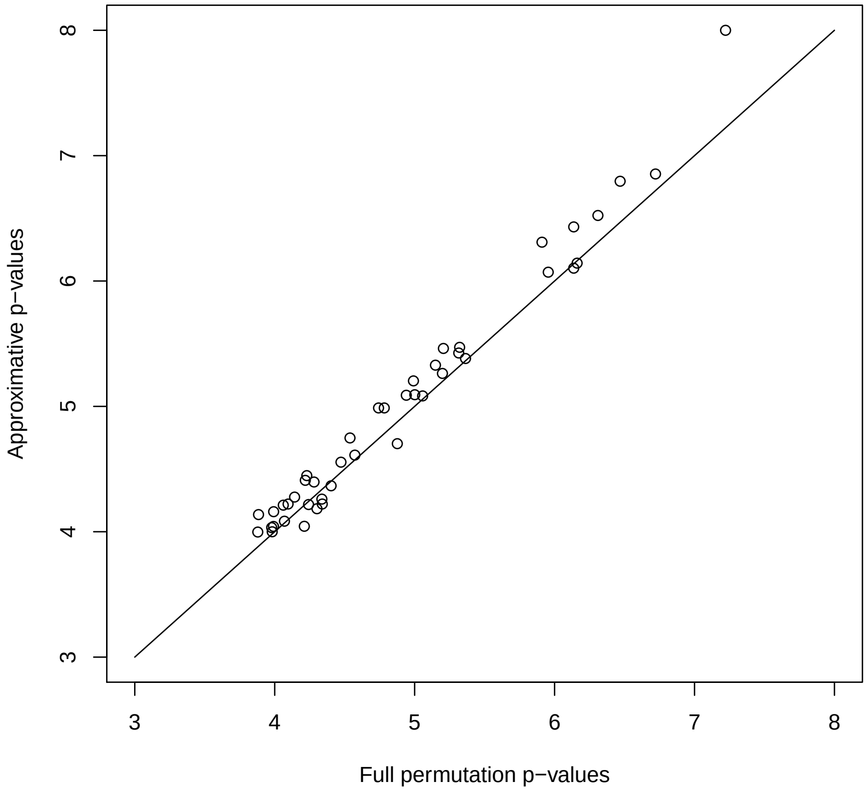

Figure S5: Comparing p-values computed on a gene by gene basis with 100000000 permutations to p-values estimated with our optional approach of non-gene-specific p-value calibration (described in the method section as Approach 2). Both p-values computations were done on the same 50 probes from the Rheumatoid Arthritis Methylation data. The probes were randomly sampled from the top 500 most significant probes. Runtime was about a week on a personal laptop. 6 probes had p-values of zero by both estimation methods. For the remaining probes the correlation between log-pvalues was 0.988. The result indicates that the probes found significantly associated using the approximation were also significant by the full permutations and vice versa.

Table S1: GEO Datasets used

ID	disease	Abv.	tissue	Data type	cases	controls
GSE63063	Alzheimer disease	AD	blood	Gene expr.	284	238
GSE99039	Parkinson	IPD	whole blood	Gene expr.	205	233
GSE73094	Crohn's disease	CD	colon/terminal ileum	Gene expr.	314	181
GSE73094	Ulcerative Colitis	UC	colon/terminal ileum	Gene expr.	181	314
GSE73094	Inflammatory Bowel disease	IBD	colon/terminal ileum	Gene expr.	374	609
GSE47862	hereditary breast cancer	-	peripheral blood	Gene expr.	158	163
GSE48091	breast cancer metastasis	-	primary cancer	Gene expr.	166	340
GSE42861	Rheumatoid Arthritis	RA	Leukocytes	Methylation	354	335
GSE74193	Schizophrenia	Sch	prefrontal cortex	Methylation	191	335
GSE80417	Schizophrenia	Sch	whole blood	Methylation	305	333
GSE73002	Breast cancer	-	serum	miRNA	1280	2686
GSE106817	Ovarian cancer	-	serum	miRNA	320	2759

Table S2: Effect of changing k in the miRNA cancer datasets

Threshold		Bonferroni			FDR ¡ 0.2		
Method	k	our test	Limma	∩	our test	Limma	∩
breast miRNA	30	480	0	0	658	1	1
breast miRNA	100	427	0	0	590	0	0
breast miRNA	200	425	0	0	580	0	0
breast miRNA	400	422	0	0	580	0	0
ovarian miRNA	30	462	58	49	1106	258	231
ovarian miRNA	100	364	5	4	984	31	31
ovarian miRNA	200	352	3	2	987	7	7
ovarian miRNA	400	361	2	2	975	6	6

Table S3: Other omics: Number of genes detected with $k = 30$

Threshold	Bonferroni			FDR ¡ 0.1		
Method	our test	Limma	∩	our test	Limma	∩
RA methylation	169	3	0	726	4	4
Schizo. cortex	67	0	0	326	0	0
Schizo. blood	266	0	0	28472	0	0
breast miRNA	483	0	0	628	0	0
ovarian miRNA	462	58	48	1012	197	179

Table S4: Comparison with Wilcoxon: Number of genes detected. $k = 100$

Threshold	Bonferroni			FDR ¡ 0.1		
Method	our test	Wilcoxon	$\cap$	our test	Wilcoxon	$\cap$
RA methylation	119	98	40	506	352	147
Schizo. cortex	22	18	6	139	85	21
Schizo. blood	75	61	7	530	242	58
breast miRNA	427	172	75	564	448	238
ovarian miRNA	364	34	22	849	310	193

Table S5: Gene expression data: Number of genes detected with $k = 100$

Threshold	Bonferroni			FDR ¡ 0.1		
Method	esa	Limma	∩	esa	Limma	∩
Alzheimer vs ctr	23	25	19	65	104	48
Parkinson	0	0	0	0	0	0
CD	2	0	0	2	0	0
UC	0	0	0	0	0	0
IBD inflammation	2	0	0	19	0	0
breast cancer	0	1	0	35	7	2
breast cancer metastasis	0	0	0	1	0	0

Table S6: Comparison with Wilcoxon. Gene expression datasets. $k = 30$

Threshold	Bonferroni			FDR ¡ 0.1		
Method	our test	Wilcoxon	$\cap$	our test	Wilcoxon	$\cap$
Alzheimer vs ctr	22	22	18	69	111	50
Parkinson	0	0	0	0	0	0
CD	2	1	1	2	3	1
UC	1	1	0	1	3	0
IBD inflammation	8	3	2	49	4	4
breast cancer	15	11	7	406	224	118
breast cancer metastasis	2	1	1	4	1	1

Table S7: Comparison of the false positive rate and the power of our test, and 11 other approaches and statistical tests. The average performance over 200 simulations with $d = 1.5$ is shown here.

	**Power**				**False discovery rate**			
Sample size n	500	650	800	1000	500	650	800	1000
Our test	0.25	0.409	0.571	0.728	0.094	0.084	0.093	0.097
COPA75	0.058	0.082	0.142	0.236	0.088	0.097	0.109	0.088
COPA9	0.054	0.106	0.17	0.26	0.088	0.076	0.099	0.108
COPA95	0.027	0.05	0.113	0.169	0.058	0.064	0.08	0.078
Outlier-sum	0.048	0.077	0.143	0.23	0.098	0.111	0.095	0.095
Wilcoxon	0.038	0.058	0.1	0.215	0.094	0.113	0.105	0.098
Kolmogorov-Smirnov	0.016	0.024	0.058	0.123	0.116	0.071	0.081	0.091
Logistic regression	0.062	0.116	0.196	0.365	0.064	0.111	0.118	0.101
ANOVA	0.062	0.116	0.196	0.365	0.064	0.111	0.118	0.101
Limma	0.068	0.126	0.208	0.377	0.092	0.121	0.12	0.107
t-test	0.068	0.126	0.208	0.375	0.092	0.121	0.119	0.106
Levene	0.02	0.037	0.068	0.118	0.08	0.089	0.103	0.097
Fisher Combination	0.174	0.302	0.454	0.673	0.21	0.191	0.209	0.188
K-Means+ chi-square	0.014	0.019	0.04	0.074	0.083	0.077	0.082	0.108

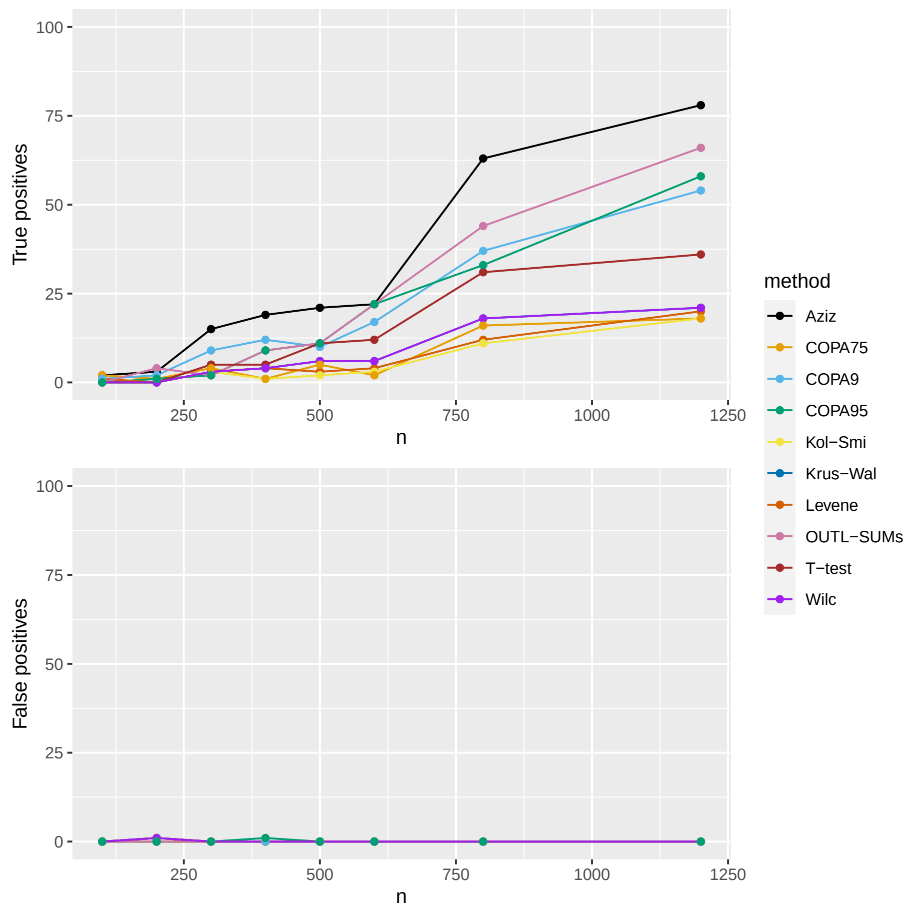

Figure S6: Power (top) and False positives (bottom) of different methods. Normal distribution and balanced setting. Simulations of 100 positive examples and 100 negative examples were performed for each sample size. $r = 0.1$ and $d = 1.5$. A p-value threshold of $\frac{0.05}{200}$ was considered. We followed the same simulation procedure as section 2.2.1 of the main paper.

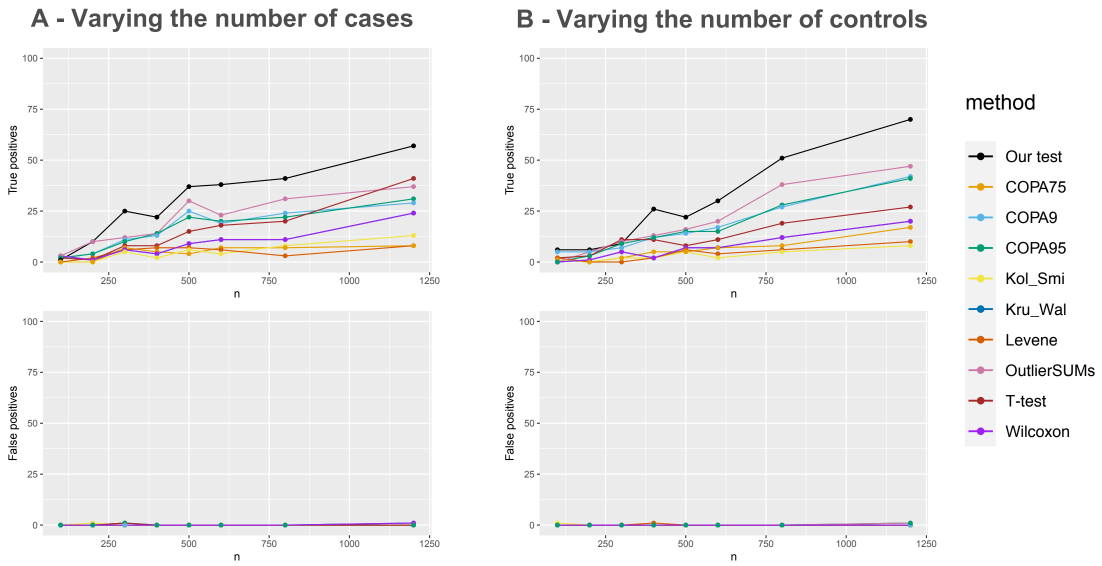

Figure S7: Power and False positives of different methods in an unbalanced case/control setting. **A** Effect on performance of varying the number of cases (fixed number of controls to 600) and **B** Effect on performance of varying the number of controls (fixed number of cases to 600). Power (top) and False positives (bottom) of different methods. Simulations of 100 positive examples and 100 negative examples were performed for each sample size. $r = 0.1$ and $d = 1.5$. A p-value threshold of $\frac{0.05}{200}$ was considered. We followed the same simulation procedure as section 2.2.1 of the main paper.

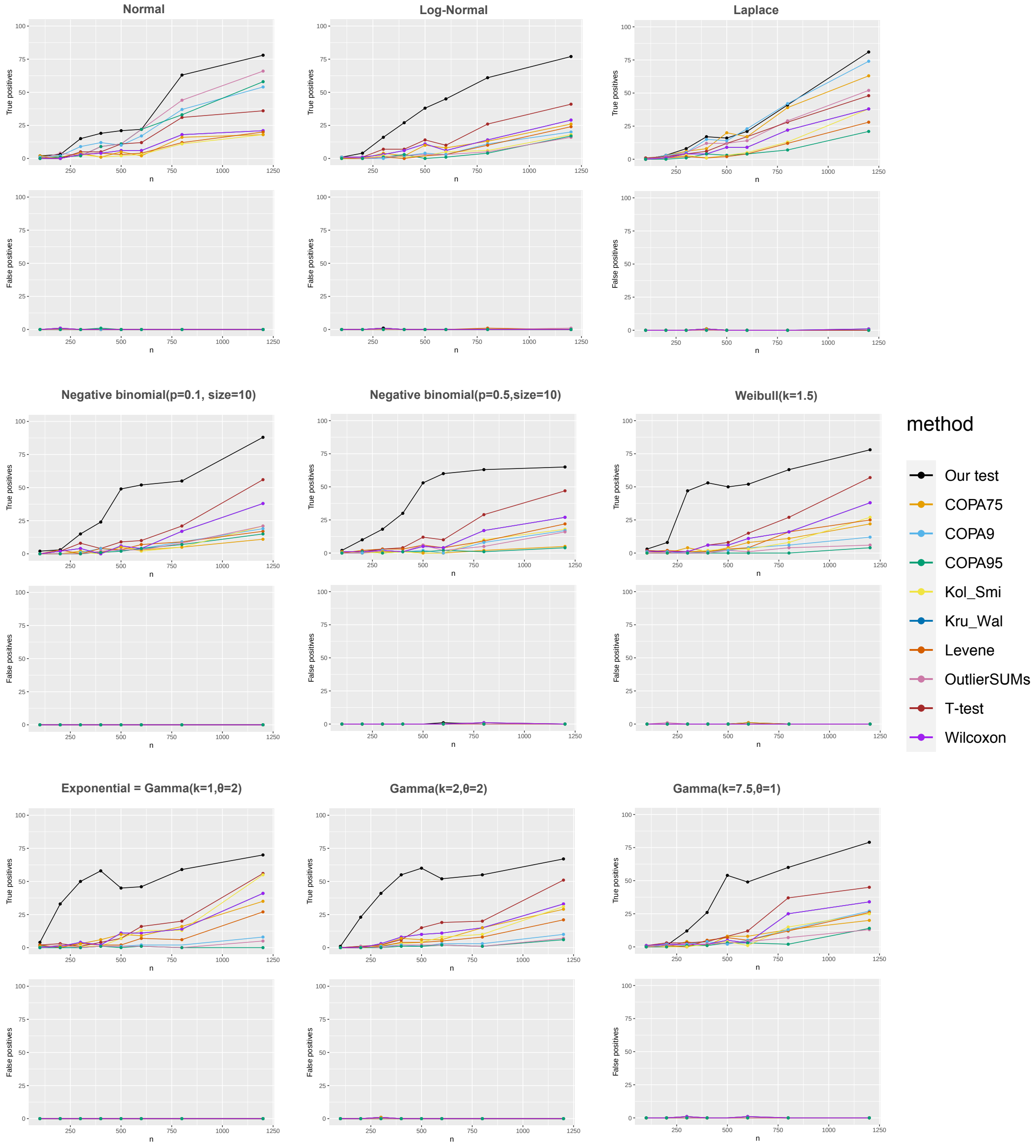

Figure S8: Performance under different data-generating probability distributions Power (top) and False positives (bottom) of different methods. Simulations of 100 positive examples and 100 negative examples were performed for each sample size. $r = 0.1$ and $d = 1.5$. A p-value threshold of $\frac{0.05}{200}$ was considered. We followed the same simulation procedure as section 2.2.1 of the main paper. No additional preprocessing was used.

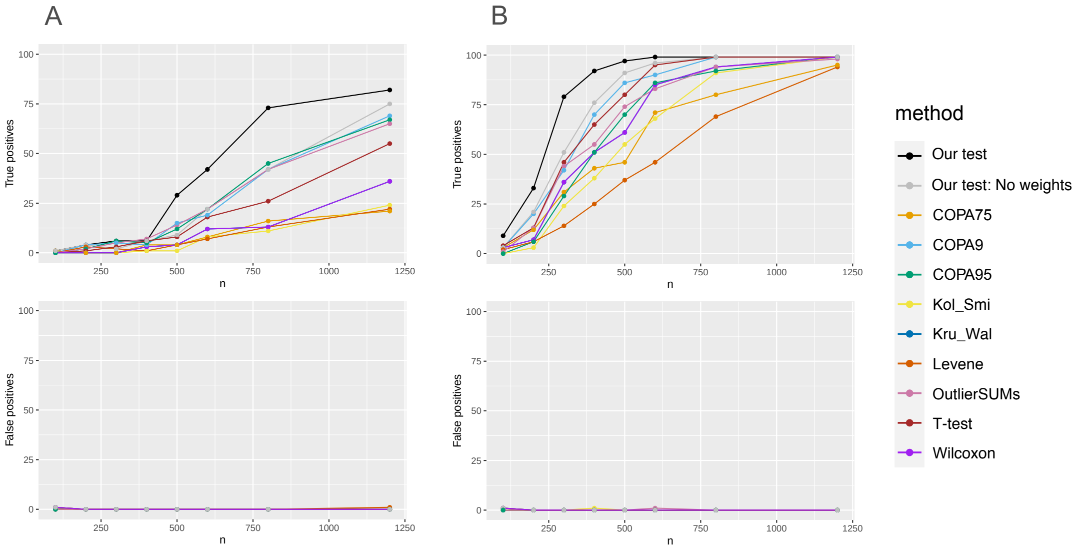

Figure S9: Effect of changing the weighting scheme for our test to all weights are equal to 1 (magnitude of aberration has no effect, only the ranking matter). **A** $r = 0.1$ **B** $r = 0.2$. Power (top) and False positives (bottom) of different methods. Simulations of 100 positive examples and 100 negative examples were performed for each sample size. $d = 1.5$. A p-value threshold of $\frac{0.05}{200}$ was considered. We followed the same simulation procedure as section 2.2.1 of the main paper.

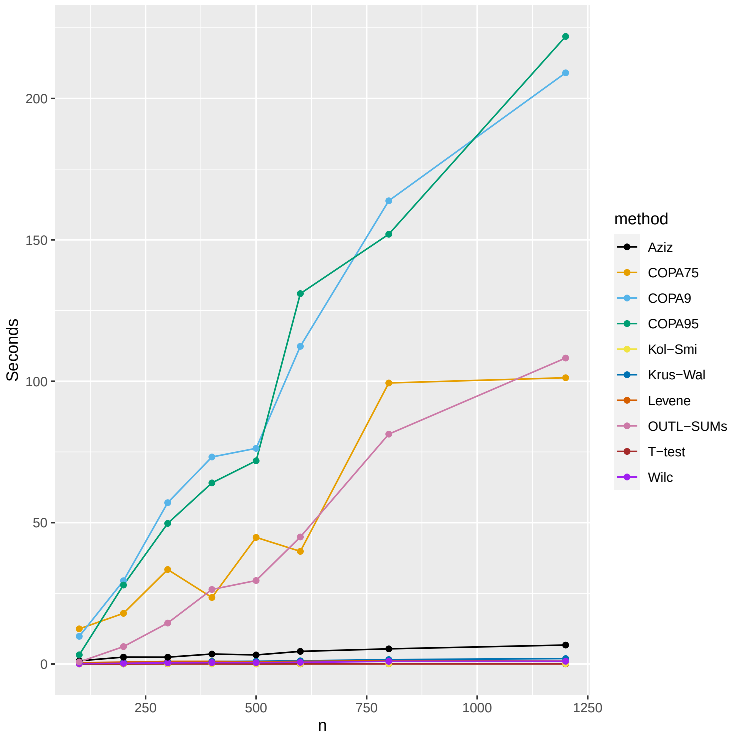

Figure S10: Running time of different methods on a personal laptop. Simulations of 100 positive examples were performed for each sample size. $r = 0.1$ and $d = 1.5$. We followed the same simulation procedure as section 2.2.1 of the main paper and Figure S6 (Gaussian).

Table S8: Cancer-control classification performance on held-out data with Area under the Precision-Recall curve (AUPRC) after feature selection by different methods. Here we vary the number of features selected for Limma and our method (first 3 columns).

Features	Top Limma features	Top Heterogeneous features	Top Het. features transformed	All features	Literature features
Ovarian Cancer n=20	0.70	0.583	**0.865**	0.696	0.503
Ovarian Cancer n=100	0.696	0.642	**0.959**	0.696	0.503
Ovarian Cancer n=300	0.695	0.743	**0.948**	0.696	0.503
Breast Cancer n=20	0.597	0.586	**0.865**	0.632	0.541
Breast Cancer n=100	0.605	0.588	**0.921**	0.632	0.541
Breast Cancer n=300	0.530	0.612	**0.965**	0.632	0.541

References

1. O. Stegle, L. Parts, M. Piipari, J. Winn, and R. Durbin, "Using probabilistic estimation of expression residuals (peer) to obtain increased power and interpretability of gene expression analyses," *Nature protocols*, vol. 7, no. 3, p. 500, 2012.
2. X. Li, Y. Kim, E. K. Tsang, J. R. Davis, F. N. Damani, C. Chiang, G. T. Hess, Z. Zappala, B. J. Strober, A. J. Scott, *et al.*, "The impact of rare variation on gene expression across tissues," *Nature*, vol. 550, no. 7675, pp. 239–243, 2017.
3. S. Sood, I. J. Gallagher, K. Lunnon, E. Rullman, A. Keohane, H. Crossland, B. E. Phillips, T. Cederholm, T. Jensen, L. J. van Loon, *et al.*, "A novel multi-tissue rna diagnostic of healthy ageing relates to cognitive health status," *Genome biology*, vol. 16, no. 1, p. 185, 2015.
4. R. Shamir, C. Klein, D. Amar, E.-J. Vollstedt, M. Bonin, M. Usenovic, Y. C. Wong, A. Maver, S. Poths, H. Safer, *et al.*, "Analysis of blood-based gene expression in idiopathic parkinson disease," *Neurology*, vol. 89, no. 16, pp. 1676–1683, 2017.
5. J. M. Peloquin, G. Goel, L. Kong, H. Huang, T. Haritunians, R. B. Sartor, M. J. Daly, R. D. Newberry, D. P. McGovern, V. Yajnik, *et al.*, "Characterization of candidate genes in inflammatory bowel disease–associated risk loci," *JCI insight*, vol. 1, no. 13, 2016.
6. S. R. Piccolo, L. M. Hoffman, T. Conner, G. Shrestha, A. L. Cohen, J. R. Marks, L. A. Neumayer, C. A. Agarwal, M. C. Beckerle, I. L. Andrulis, *et al.*, "Integrative analyses reveal signaling pathways underlying familial breast cancer susceptibility," *Molecular systems biology*, vol. 12, no. 3, 2016.
7. A. Lundberg, L. S. Lindström, J. C. Harrell, C. Falato, J. W. Carlson, P. K. Wright, T. Foukakis, C. M. Perou, K. Czene, J. Bergh, *et al.*, "Gene expression signatures and immunohistochemical subtypes add prognostic value to each other in breast cancer cohorts," *Clinical Cancer Research*, vol. 23, no. 24, pp. 7512–7520, 2017.
8. A. E. Jaffe, Y. Gao, A. Deep-Soboslay, R. Tao, T. M. Hyde, D. R. Weinberger, and J. E. Kleinman, "Mapping dna methylation across development, genotype and schizophrenia in the human frontal cortex," *Nature neuroscience*, vol. 19, no. 1, p. 40, 2016.
9. E. Hannon, E. Dempster, J. Viana, J. Burrage, A. R. Smith, R. Macdonald, D. St Clair, C. Mustard, G. Breen, S. Therman, *et al.*, "An integrated genetic-epigenetic analysis of schizophrenia: evidence for co-localization of genetic associations and differential dna methylation," *Genome biology*, vol. 17, no. 1, p. 176, 2016.
10. Y. Liu, M. J. Aryee, L. Padyukov, M. D. Fallin, E. Hesselberg, A. Runarsson, L. Reinius, N. Acevedo, M. Taub, M. Ronninger, *et al.*, "Epigenome-wide association data implicate dna methylation as an intermediary of genetic risk in rheumatoid arthritis," *Nature biotechnology*, vol. 31, no. 2, p. 142, 2013.
11. A. Shimomura, S. Shiino, J. Kawauchi, S. Takizawa, H. Sakamoto, J. Matsuzaki, M. Ono, F. Takeshita, S. Niida, C. Shimizu, *et al.*, "Novel combination of serum microrna for detecting breast cancer in the early stage," *Cancer science*, vol. 107, no. 3, pp. 326–334, 2016.
12. A. Yokoi, J. Matsuzaki, Y. Yamamoto, Y. Yoneoka, K. Takahashi, H. Shimizu, T. Uehara, M. Ishikawa, S.-i. Ikeda, T. Sonoda, *et al.*, "Integrated extracellular microrna profiling for ovarian cancer screening," *Nature communications*, vol. 9, no. 1, pp. 1–10, 2018.
13. B. Phipson and G. K. Smyth, "Permutation p-values should never be zero: calculating exact p-values when permutations are randomly drawn," *Statistical applications in genetics and molecular biology*, vol. 9, no. 1, 2010.
